# Clinical features, genotypes, and geographic distribution of 238 Latin American CGD patients

**DOI:** 10.70962/jhi.20250033

**Published:** 2025-10-06

**Authors:** Tiago Santos de Oliveira, Ranieri Coelho Salgado, Lillian Nunes Gomes, Carolina Sanchez Aranda, Janáira Fernandes Severo Ferreira, Ekaterini Simões Goudouris, Fernanda Pinto Mariz, Maria Luiza Oliva Alonso, Solange Oliveira Rodrigues Valle, Luiz Fernando Bacarini Leite, Ana Carolina da Matta Ain, Pérsio Roxo-Junior, Marília Montanaro, Flávia Alice Timburibá de Medeiros Guimarães, Simone de Castro Resende Franco, Herberto José Chong-Neto, Nelson Augusto Rosario Filho, Daniélli Christinni Bichuetti-Silva, Vera Maria Dantas, Jackeline Motta Franco, Viviane Guimaro Mendes Barreto Resende, Simone Soares Lima, Dorcas Lamounier Costa, Mayra De Barros Dorna, Antônio Carlos Pastorino, Beni Morgenstern, Vera Esteves Vagnozzi Rullo, Constantino Giovanni Braga Cartaxo, Naiade R. de Sá, Irma Cecília Douglas Paes Barreto, Nancy Viegas Chen Lobo, Flavia Amendola Anisio de Carvalho, Monica Soares de Souza, Gesmar Rodrigues Silva Segundo, Dewton de Moraes Vasconcelos, Juliana Folloni Fernandes, Gislene Santana Tusani, Carolina Cardoso de Mello Prando, Ana Paula Zaninelli Diniz Iwamura, Arturo López-Larios, Ana Jocelyn Carmona Vargas, Ana Paola Macías-Robles, Maria Edith Gonzalez Serrano, Sara Elva Espinosa-Padilla, Ana Karen Peñafiel Vicuña, Aidé Tamara Staines-Boone, Omar J. Saucedo-Ramírez, Miguel García Domínguez, Maria R. Canseco-Raymundo, Federico Saracho-Weber, Oscar Zavaleta Martínez, Susana García-Pavón-Osorio, Rogelio Guzmán Cotaya, Francisco J. Espinosa-Rosales, Roselia Ramírez-Rivera, María de la Luz H. García-Cruz, Carmen Zarate-Hernández, Lizbeth Blancas-Galicia, Alejandra King, Oscar Porras, Lorena Regairaz, Alejandra Lampugnani, Celia Martínez de Cuellar, Juan Carlos Aldave Becerra, Liz Eliana Veramendi Espinoza, Cecilia Montenegro, Magdalena Schelotto, Virginia Patiño, Mariane Cunha de Oliveira, Antonio Condino-Neto

**Affiliations:** 1 Department of Immunology, Institute of Biomedical Sciences, University of São Paulo, São Paulo, Brazil; 2 Department of Pediatrics, Paulista School of Medicine, Federal University of São Paulo, São Paulo, Brazil; 3 Albert Sabin Children’s Hospital, Fortaleza, Brazil; 4 Federal University of Rio de Janeiro, Rio de Janeiro, Brazil; 5 Santa Casa de Misericórdia de São Paulo, São Paulo, Brazil; 6 Taubaté University - Municipal University Hospital of Taubaté, Taubaté, Brazil; 7 Faculty of Medicine of the University of São Paul, Ribeirão Preto, Brazil; 8 Brasilia Mother and Child Hospital, Brasília, Brazil; 9 Division of Allergy and Immunology, Complexo Hospital de Clínicas da Universidade Federal do Paraná, Curitiba, Brazil; 10 Federal University of Goias, Goiania, Brazil; 11 Federal University of Rio Grande do Norte, Natal, Brazil; 12 Federal University of Sergipe, Aracajú, Brazil; 13 Regional Hospital of Mato Grosso do Sul, Campo Grande, Brazil; 14 Lucidio Portela Children’s Hospital, Teresina, Brazil; 15 Children’s Institute, Faculty of Medicine of the University of São Paulo, São Paulo, Brazil; 16 Santos Faculty of Medical Sciences, Lusíada University Center, Santos, Brazil; 17 Department of Pediatrics and Genetics, Federal University of Paraíba, João Pessoa, Brazil; 18 University Center of the State of Pará, Belém, Brazil; 19 Private Office, Maceió, Brazil; 20 Fernandes Figueira Institute, Fiocruz, Rio de Janeiro, Brazil; 21 Head of Pediatric Immunoallergy at the Hospital Federal dos Servidores do Estado do Rio de Janeiro, Rio de Janeiro, Brazil; 22 Federal University of Uberlandia, Uberlândia, Brazil; 23 Children’s Cancer Treatment Institute, Faculty of Medicine of the University of São Paulo, São Paulo, Brazil; 24 Insituto de Pesquisa Pelé Pequeno Príncipe, Faculdades Pequeno Príncipe, Hospital Pequeno Príncipe, Curitiba, Brazil; 25 Unidad de Inmunología Clínica, Hospital CIMA, Hermosillo, Mexico; 26 Departamento de Enfermedades Infecciosas, Instituto Nacional de Pediatria, Mexico city, Mexico; 27 Departamento de Alergia e Inmunología Clínica Pediátrica, UMAE, CMNO, Guadalajara, Mexico; 28 Immunodeficiencies Laboratory, Instituto Nacional de Pediatria, Mexico City, Mexico; 29 Departamento de Inmunología, UMAE, Monterrey, Mexico; 30 Departamento de Alergia, Hospital infantil de México Federico Gómez, Mexico City, Mexico; 31 Hospital Pediátrico de Sinaloa “Dr. Rigoberto Aguilar Pico,” Culiacan, México; 32 Departamento de Alergia e Inmunología Clínica, UMAE, “LA RAZA,” Mexico City, México; 33 Departamento de Enseñanza, Hospital Infantil de Especialidades, Mexico City, México; 34 Allergy and Immunology Department, Hospital Materno Infantil, Instituto de Seguridad Social del Estado de México y Municipios, Toluca, Mexico; 35 High Specialty Naval Hospital, Mexico City, Mexico; 36 Chavitos Clinic, Merida, Mexico; 37 Fundación Mexicana para Niños y Niñas con Inmunodeficiencias, Mexico City, Mexico; 38 Departamento de Pediatría, Hospital de Especialidades del Niño y la Mujer, “Dr Felipe Nuñez Lara,” Querétaro, Mexico; 39 Departamento de Otorrinolaringologia y Cirugía de Cabeza y Cuello, Instituto Nacional de Enfermedades Respiratorias, Mexico city, México; 40 Centro Regional de Alergia e Inmunología Clínica, Hospital Universitario “Dr. José Eleuterio González,” Universidad Autónoma de Nuevo León, Monterrey, México; 41 Facultad de Medicina, Universidad de Chile, Santiago, Chile; 42 Hospital Nacional de Ninos “Dr Carlos Saenz Herrera,” San Jose, Costa Rica; 43 Unidad de Inmunología, Hospital de Niños Sor María Ludovica, Buenos Aires, Argentina; 44 Hospital Rawson, San Juan, Argentina; 45 Universidad Nacional de Asunción, San Lorenzo, Paraguay; 46 Division of Allergy and Immunology, Hospital Nacional Edgardo Rebagliati Martins, Lima, Peru; 47 Oficina General de Investigación y Transferencia Tecnológica, Instituto Nacional de Salud, Lima, Peru; 48 Centro Hospitalario Pereira Rossell, Montevideo, Uruguay; 49 Centro Universitário das Faculdades Metropolitanas Unidas, São Paulo, Brazil

## Abstract

Chronic granulomatous disease (CGD) is an inborn error of immunity (IEI) caused by mutations in genes encoding components of the NADPH oxidase complex, leading to defective microbial killing and increased susceptibility to infections. This study analyzed clinical, genetic, and geospatial data from 238 CGD patients across eight Latin American countries. Genetic variants were identified in 141 patients (59%), with XL-CGD being the most common form (77%). Pneumonia (80%), lymphadenopathy (63%), and skin infections (55.5%) were frequent, with bacteria and fungi, such as *Staphylococcus aureus*, *Aspergillus* spp., and mycobacteria, as major pathogens. Antimicrobial prophylaxis was widely used, while IFN-γ was mainly prescribed in Mexico, mainly in cases of classic CGD (XL-CGD). Hematopoietic stem cell transplantation (HSCT) did not improve survival compared to prophylaxis. The leading cause of death was infection, particularly pneumonia and sepsis. XL-CGD patients had worse survival outcomes. The study highlights the need for improved genetic diagnosis, newborn screening, regional treatment guidelines, and expanded access to HSCT.

## Teaser

This epidemiological study describes CGD in Latin America. It highlights high infection risks, limited treatment options, and poor survival rates, especially for males with XL-CGD. The findings emphasize the need for better diagnosis, newborn screening, regional treatment guidelines, and expanded access to effective therapies.

## Introduction

Chronic granulomatous disease (CGD) is a heterogeneous inborn error of immunity (IEI) caused by defects in the nicotinamide adenine dinucleotide phosphate (NADPH) oxidase complex of phagocytes, leading to varying levels of impairment in the oxidative burst in response to stimuli, a relevant mechanism for effective microbial killing (1). Due to this defect, CGD is characterized by excessive inflammation and recurrent and severe infections caused mainly by extracellular pyogenic bacteria, intracellular bacteria and fungi (particularly catalase producers), challenging inflammatory manifestations, and autoimmunity (2).

Phagocyte NADPH oxidase is composed of five main subunits: Two proteins anchored to the plasma membrane or phagosomal/phagolysosomal membrane that form the cytochrome b_558_, and three cytoplasmic proteins that migrate and anchor to cytochrome b_558_ to form the NADPH oxidase complex upon stimulation (2). CGD can occur in the X-linked (XL) form when pathogenics variants affect the *CYBB* gene (OMIM# 306400), (Xp21.1), which encodes the 91-kDa β subunit of cytochrome b_558_, gp91^phox^, and in the autosomal recessive (AR) form when mutations occur in the *CYBA* (16q24), *NCF1* (7q11.23), *NCF2* (1q25), and *NCF4* (22q13.1) genes (the latter associated with p40^phox^ deficiency, a CGD-like but distinct phagocyte disorder), and the more recently identified *CYBC1* (17q25.3) gene (1, 2), (OMIM# 233690, 233700, 233710, 613960, 618935, respectively), which encode p22^phox^ (α subunit of cytochrome b_558_), p47^phox^, p67^phox^, p40^phox^, and EROS, respectively (3, 4, 5, 6, 7).

The main manifestations of CGD include pneumonia (PNM), lymphadenitis, skin infections with or without abscesses, deep abscesses (especially hepatic), hepatosplenomegaly, osteomyelitis, and infectious or inflammatory gastroenteropathies. The latter, in a state of hyperinflammation, can also present as chronic colitis with the formation of obstructive granulomas (also observed in the genitourinary tract), making the gastrointestinal tract the most affected by inflammatory processes (8, 9, 10, 11, 12, 13, 14, 15, 16). The most commonly isolated pathogens in CGD include *Staphylococcus* species, especially *Staphylococcus aureus*, *Burkholderia* spp., *Serratia* spp., *Nocardia* (more common in temperate countries), *Aspergillus* spp., *Mycobacterium tuberculosis*, and *Bacillus Calmette-Guérin* (BCG) infections, in countries where tuberculosis (TB) is endemic and BCG vaccination is mandatory (8, 9, 10, 11, 12, 13, 14, 15, 16, 17, 18, 19, 20). Treatment involves using antibiotics and antifungals for active infections and corticosteroids for challenging inflammatory processes. However, prophylaxis with cotrimoxazole plus azole antifungals provides a better quality of life and survival for these patients, sometimes also benefiting from the use of interferon-gamma (IFN-γ) (21, 22). The only curative therapy currently is hematopoietic stem cell transplantation (HSCT) (23, 24, 25).

The estimated incidence of CGD is around 1:200,000–1:250,000 live births in the United States (9). Still, it varies considerably across different geographic regions: 1:450,000 in Sweden, 1:300,000 in Japan, 1:218,000 among Israeli Jews, and the highest estimated incidence among Israeli Arabs at 1:100,000 (11, 26). Generally, XL-CGD accounts for about 60–70% of cases, followed by p47^phox^ defects in up to 30% of cases. However, the AR form may occur at a frequency similar to or even higher than the XL form in countries with high consanguinity rates (11, 26). The true incidence of CGD in Latin American countries remains largely unknown.

Studies on CGD in Latin America are mostly confined to case reports and clinical studies with few patients, rarely exceeding a dozen (27), with few more representative clinical-epidemiological studies (8, 13, 28). As the literature shows, the clinical characteristics of CGD patients in developing countries differ from those classically reported in patients from developed countries. Therefore, clinical-epidemiological studies are relevant to better understanding CGD patients in this region.

This study summarizes the clinical, epidemiological, and genetic characteristics of 238 patients with CGD from eight Latin American countries, making it the most extensive clinical-epidemiological study on CGD in Latin America to date.

## Results

### Clinical characteristics and geographic distribution of 238 CGD patients from Latin America

**Figure 1.**
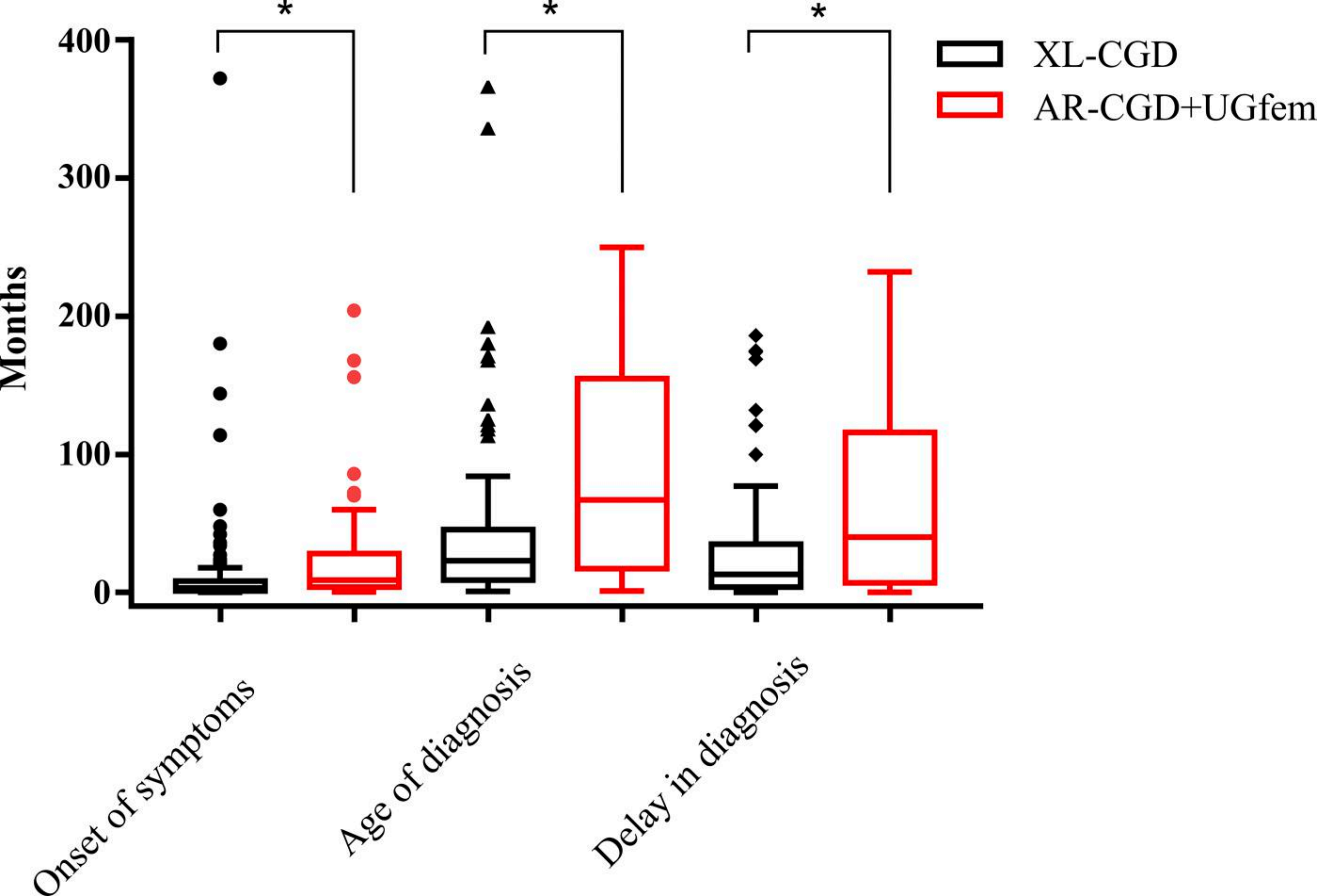
**Comparison between XL- and AR-CGD regarding onset of symptoms, age of diagnosis, and delay in diagnosis.** Patients with XL-CGD have earlier onset of symptoms, as well as age of diagnosis and delay in diagnosis when compared to patients with AR-CGD (AR-CGD + UGfem patients). * Mann–Whitney test (P < 0.001).

A total of 238 patients from 210 unrelated families (109 familial cases, 22 consanguineous families) at 53 pediatric healthcare centers in Mexico (*n* = 118; 50%), Brazil (*n* = 96; 40%), Chile (*n* = 6; 2%), Costa Rica (*n* = 5; 2%), Argentina (*n* = 4; 2%), Paraguay (*n* = 4; 2%), Peru (*n* = 3; 1%), and Uruguay (*n* = 2; 1%) were included in the study. Among the 238 patients, 196 were male and 42 were female. Of the male patients, 107 had confirmed XL-CGD and 12 had AR-CGD. Among the females, 20 had AR-CGD and two were likely XL-CGD carriers with clinical manifestations, possibly due to skewed X-inactivation. The remaining patients had undefined CGD subtype (unknown genotype [UG]-CGD). The patients were diagnosed between 1976 and 2021, with 229 diagnosed by dihydrorhodamine-1,2,3 (DHR) (including confirmations by nitroblue tetrazolium [NBT]), eight by NBT, and one with a genetic diagnosis of XL-CGD and a carrier mother (P170 and P145, respectively). Table 1 summarizes the demographic characteristics of the patients, and more detailed individual descriptions are available in Table 2 and Table S1. The median age at presentation of the first clinical manifestation of CGD was 5 mo (range: 0.1–336 mo). The median age at diagnosis was 2 years and 2 mo (range: 0.3–366 mo), with a median diagnosis delay of 1 year and 2 mo (range: 0.1–232 mo). 173 patients (73%) were diagnosed before the age of 6 years. For statistical purposes, female patients with UGs, all with AR-CGD phenotype, were included in the AR-CGD group, forming the AR-CGD + UG female (UGfem) group with 52 individuals, allowing the following analyses. The median age at onset of clinical manifestations for patients with XL-CGD was 3 mo (range, 0.1–336 mo), which was earlier than the median age for patients with AR-CGD, at 8.5 mo (range: 0.25–204 mo), and for the AR-CGD + UGfem group, at 9 mo (range: 0.25–204 mo) (P < 0.001). The date or age of the first clinical manifestation was obtained for 225 patients. A similar pattern was observed in the median age at diagnosis: patients with XL-CGD were diagnosed at a median age of 23 mo (range: 0.6–366 mo), compared to 60 mo (range: 1–235 mo) for AR-CGD and 67 mo (range: 1–250 mo) for the AR-CGD + UGfem group (P < 0.001). Date or age at diagnosis was available for 229 patients. Again, the median diagnosis delay for XL-CGD patients was shorter, at 13 mo (range: 0.1–186 mo), compared to 37 mo (range: 0.1–227 mo) for AR-CGD and 40 mo (range: 0.1–232 mo) for the AR-CGD + UGfem group (P < 0.001) (Fig. 1).

**Table 1.** Demographic characteristics of Latin American patients with CGD (*n* = 238)

​	Total	XL-CGD	AR-CGD	AR-CGD + UGfem	UG-CGD
No. of patients, *n* (%)	238 (100)	109 (46)	32 (13)	52 (29)	97 (41)
Family, *n*	210	94	28	47	88
Consanguinity, *n* (%)	22 (10)	3 (3)	13 (46)	14 (30)	6 (6.2)
Family history of CGD, *n* (%)	41 (19)	22 (23)	5 (18)	8 (17)	14 (14.4)
Outcome					
Undergoing HSTC, *n* (%)	53 (22)	22 (20)	5 (16)	8 (15)	26 (26.8)
HSCT deceased, *n* (%)	16 (30)	7 (31)	2 (40)	2 (25)	7 (27)
Deceased, *n* (%)	80 (34)	43 (54)*	4 (5)	12 (15)*	33 (34)
Age of death	66m (1m–31y2m)	60.5m (5m–31y2m)	133.5m (2 years–19y6m)	88m (1y5m–23y)	75m (1m–25y)

General demographic characteristics of Latin American CGD patients by genotype/phenotype (XL-CGD, AR-CGD, AR-CGD + UGfem, and UG-CGD) and total. For statistical purposes, UGfem were included in the AR-CGD group, forming the group AR-CGD + UGfem. y, year; m, month. *χ^2^ test (P = 0.041).

**Table 2.** Genetic characterization and clinical outcome of patients with CGD in Latin America (*n* = 141)

ID	Fam.	Country	Sex	Gene	Site	Pathogenic variant	Protein	Type	Zygo.	Express.	Outcome	Ref.
P1	A	BRA	M	*CYBB*	ex. 9	c.1158delG	p.Trp380*	*Nonsense*	Hemi	−	Alive	ND
P3	Csi	BRA	M	*CYBB*	ex. 2	c.125C>A	p.Thr42Lis	*Missense*	Hemi	−	Dead	(3)
P5	Dsi	BRA	M	*NCF1*	ex. 2	c.75_76delGT	p.Tyr26Hisfs*26	*Deletion*	Homo	A47^0^	Alive	(4)
P6	Dsi	BRA	F	*NCF1*	ex. 2	c.75_76delGT	p.Tyr26Hisfs*26	*Deletion*	Homo	A47^0^	Dead	(4)
P16	N	BRA	F	*NCF1*	ex. 2	c.75_76delGT	p.Tyr26Hisfs*26	*Deletion*	Homo	A47^0^	Alive	(4)
P17	O	BRA	M	*CYBB*	ex. 9	c.1022C>T	p.Thr341Ile	*Missense*	Hemi	X91^−^	Alive	(3, 29)
P18	P	BRA	M	*CYBB*	ex. 6	c.665A>T	p.His222Leu	*Missense*	Hemi	X91^?^	Dead	(3)
P19	Qc	BRA	M	*CYBB*	ex. 3	c.217C>T	p.Arg73*	*Nonsense*	Hemi	X91^0^	Dead	(3, 29)
P20	Rsi	BRA	M	*CYBB*	ex. 1	c.12_15delGGCT	p.Ala5*	*Nonsense*	Hemi	−	Dead	(30)
P21	Rsi	BRA	M	*CYBB*	ex. 1	c.12_15delGGCT	p.Ala5*	*Nonsense*	Hemi	−	Dead	(30)
P23	Qc	BRA	M	*CYBB*	ex. 3	c.217C>T	p.Arg73*	*Nonsense*	Hemi	X91^0^	Alive	(3,29)
P25	Qc	BRA	M	*CYBB*	ex. 3	c.217C>T	p.Arg73*	*Nonsense*	Hemi	X91^0^	Alive	(3, 29)
P27	V	BRA	M	*CYBB*	ex. 5	c.375G>A	p.Trp125*	*Nonsense*	Hemi	X91^?^	Alive	(3)
P29	X	BRA	M	*CYBB*	ex. 7	c.752G>A	p.Trp251*	*Nonsense*	Hemi	X91^0^	Dead	(3, 29)
P33	AB	BRA	F	*NCF1*	ex. 2	c.75_76delGT	p.Tyr26Hisfs*26	*Deletion*	Homo	A47^0^	Alive	(4)
P35	AD	BRA	M	*CYBB*	ex. 8	c.868C>T	p.Arg290*	*Nonsense*	Hemi	X91^0^	Alive	(3, 29)
P39	AH	BRA	M	*CYBB*	ex. 5	c.430C>T	p.Leu144Phe	*Missense*	Hemi	X91^?^	Alive	ND
P40	AI	BRA	M	*CYBB*	ex. 7	c.676C>T	p.Arg226*	*Nonsense*	Hemi	X91^0^	Alive	(3, 29)
P41	AJ	BRA	M	*CYBB*	ex. 13	c.1679delG	p.Gly560Glufs*17	*Deletion*	Hemi	X91^0^	Alive	(3)
P43	AL	BRA	F	*NCF1*	ex. 2	c.75_76delGT	p.Tyr26Hisfs*26	*Deletion*	Homo	A47^0^	Alive	(4)
P47	AP	BRA	M	*CYBB*	ex. 5	c.388C>T	p.Arg130*	*Nonsense*	Hemi	X91^0^	Alive	(3, 29)
P48	AQ	BRA	M	*CYBB*	ex. 9	c.1140G>A	p.Trp380*	*Nonsense*	Hemi	X91^0^	Alive	(3)
P50	ASsi	BRA	F	*NCF1*	ex. 2	c.75_76delGT	p.Tyr26Hisfs*26	*Deletion*	Homo	A47^0^	Alive	(4)
P51	ASsi	BRA	F	*NCF1*	ex. 2	c.75_76delGT	p.Tyr26Hisfs*26	*Deletion*	Homo	A47^0^	Alive	(4)
P52	AT	BRA	M	*CYBB*	ex. 13	c.1609T>C	p.Cys537Arg	*Missense*	Hemi	X91^+^	Alive	(3, 29)
P60	AZ	BRA	F	*CYBA*	ex. 6/ex. 5	c.472_484del/c.399delC	p.Pro160Alafs*27/p.Ile134Serfs*57	*Deletion*/*Deletion*	Comp	A22^?^/A22^?^	Alive	(4)
P62	BAc	BRA	M	*CYBB*	ex. 7	c.754G>T	p.Gly252*	*Nonsense*	Hemi	X91^?^	Alive	(3)
P63	BB	BRA	M	*CYBB*	del. ex. 1_13	del. ex. 1_13	−	Large deletion (≥1 ex.)	Hemi	X91^0^	Alive	ND
P66	BE	BRA	F	*NCF1*	ex. 2	c.75_76delGT	p.Tyr26Hisfs*26	*Deletion*	Homo	A47^0^	Alive	(4)
P69	BH	BRA	F	*NCF1*	ex. 2	c.75_76delGT	p.Tyr26Hisfs*26	*Deletion*	Homo	A47^0^	Alive	(4)
P70	BI	BRA	M	*CYBB*	del. ex. 5	c.483G>A	del. ex. 5 (p.Lys161 = )	*Splicing*	Hemi	X91^0^	Alive	(3)
P71	BJ	BRA	M	*CYBB*	ex. 5	c.376T>C	p.Cis126Arg	*Missense*	Hemi	X91^?^	Alive	(31)
P73	BL	BRA	F	*NCF1*	ex. 2	c.75_76delGT	p.Tyr26Hisfs*26	*Deletion*	Homo	A47^0^	Alive	(4)
P75	BN	BRA	M	*CYBB*	ex. 9	c.1010G>A	p.Trp337*	*Nonsense*	Hemi	X91^0^	Alive	(3, 29)
P77	BP	BRA	M	*CYBB*	del. ex. 3	c.252G>A	del. ex. 3 (p.Ser48_Ala84del)	*Splicing*	Hemi	X91^0^	Alive	(3, 29)
P97	CF	MEX	M	*CYBB*	ex. 3	c.217C>T	p.Arg73*	*Nonsense*	Hemi	X91^0^	Dead	(3, 29)
P98	CG	MEX	M	*CYBB*	ex. 13	−	p.Ile532*	*Nonsense*	Hemi	X91^0^	Alive	(13)
P101	CJ	MEX	M	*NCF1*	ex. 2	c.75_76delGT	p.Tyr26Hisfs*26	*Deletion*	Homo	A47^0^	Alive	(4)
P102	CK	MEX	M	*CYBB*	ex. 6	c.626A>G	p.His209Arg	*Missense*	Hemi	X91^0^	Alive	(13)
P103	CL	MEX	M	*CYBB*	ex. 9	c.987C>A	p.Cys329*	*Nonsense*	Hemi	X91^?^	Dead	(3)
P104	CM	MEX	M	*CYBB*	ex. 12	c.1499A>T	p.Asp500Val	*Missense*	Hemi	X91^+^	Alive	(32)
P105	CN	MEX	M	*CYBB*	ex. 6	c.602dup	p.Tyr201*	*Insertion*	Hemi	X91^0^	Dead	(13, 33)
P107	CPc	MEX	M	*CYBA*	ex. 1	c.4_24del21	p.Gly2_Met8del	*Deletion*	Homo	A22^0^	Alive	(13)
P109	CR	MEX	M	*CYBB*	ex. 12	c.1545del	p.Trp516Glyfs*17	*Deletion*	Hemi	X91^?^	Dead	(13)
P110	CSn	MEX	M	*CYBB*	ex. 9	c.1016C>A	p.Pro339His	*Missense*	Hemi	X91^0^	Dead	(3, 13, 29)
P111	CT	MEX	M	*CYBB*	ex. 8	c. 850_851delAG	p.Arg84Valfs*63	*Deletion*	Hemi	X91^0^	Dead	(13, 33)
P112	CU	MEX	M	*CYBB*	ex. 2	c.83G>A	p.Trp28*	*Nonsense*	Hemi	X91^0^	Alive	(3)
P113	CVsi	MEX	M	*CYBB*	ex. 7	c.676C>T	p.Arg226*	*Nonsense*	Hemi	X91^0^	Dead	(3, 13, 29)
P114	CVsi	MEX	M	*CYBB*	ex. 7	c.676C>T	p.Arg226*	*Nonsense*	Hemi	X91^0^	Alive	(3, 13, 29)
P115	CWsi	MEX	F	*NCF1*	ex. 2	c.75_76delGT	p.Tyr26Hisfs*26	*Deletion*	Homo	A47^0^	Alive	(4)
P116	CWsi	MEX	F	*NCF1*	ex. 2	c.75_76delGT	p.Tyr26Hisfs*26	*Deletion*	Homo	A47^0^	Alive	(4)
P117	CX	MEX	F	*CYBA*	in. 5	c.370-1G>A	Splicing site	*Splicing*	Homo	A22^0^	Alive	(13)
P118	CY	MEX	F	*NCF1*	ex. 2	c.75_76delGT	p.Tyr26Hisfs*26	*Deletion*	Homo	A47^0^	Alive	(4)
P119	CZ	MEX	M	*CYBB*	ex. 6	c.616T>C	p.Trp206Arg	*Missense*	Hemi	X91^?^	Alive	ND
P120	DA	MEX	M	*CYBB*	ex. 4	c.277C>T	p.Gln93*	*Nonsense*	Hemi	X91^?^	Dead	(8)
P124	DEsi	MEX	M	*CYBB*	ex. 5	c.425_426delCT	p.Ser142*	*Nonsense*	Hemi	X91^0^	Alive	(3)
P126	DGu	MEX	M	*CYBB*	ex. 9	c.978delT	p.Phe326Leufs*17	*Deletion*	Hemi	X91^?^	Dead	ND
P127	DEsi	MEX	M	*CYBB*	ex. 5	c.425_426delCT	p.Ser142*	*Nonsense*	Hemi	X91^0^	Alive	(3)
P135	DN	MEX	M	*CYBB*	ex. 9	c.1019T>C	p.Phe340Ser	*Missense*	Hemi	X91^?^	Dead	ND
P136	DOsi	MEX	M	*CYBB*	ex. 11	c.1447T>C	p.Trp483Arg	*Missense*	Hemi	X91^?^	Dead	(8)
P137	DP	MEX	M	*CYBB*	ex. 9	c.1011G>A	p.Trp337*	*Nonsense*	Hemi	X91^0^	Dead	(4)
P139	DR	MEX	M	*CYBB*	ex. 12	c.1571C>T	p.Ala524Val	*Missense*	Hemi	X91^0^	Dead	(3)
P141	DT	MEX	M	*CYBB*	ex. 9	c.1006G>T	p.Glu336*	*Nonsense*	Hemi	X91^0^	Dead	(3, 29)
P142	DU	MEX	M	*CYBB*	ex. 1	c.12G>A	p.Trp4*	*Nonsense*	Hemi	X91^0^	Dead	(3, 29)
P144	DW	MEX	M	*CYBB*	in. 6	c.675-12T>G	Splicing site	*Splicing*	Hemi	X91^?^	Dead	(13, 33)
P145	DXm	MEX	F	*CYBB*	ex. 7	c.676C>T	p.Arg226*	*Nonsense*	Hetero	X91^0^ #	Alive	(34)
P146	DY	MEX	M	*CYBB*	ex. 1	c.12G>A	p.Trp4*	*Nonsense*	Hemi	X91^0^	Dead	(3, 29)
P147	DZsi	MEX	M	*CYBB*	del. ex. 1_13	Del. ex. 1_13	−	Large deletion (≥1 ex.)	Hemi	X91^0^	Dead	(13)
P148	DOsi	MEX	M	*CYBB*	ex. 11	c.1447T>C	p.Trp483Arg	*Missense*	Hemi	X91^?^	Alive	(8)
P149	EA	MEX	M	*CYBB*	ex. 7	c.722_726delTAACA	p.Ile241Serfs*3	*Deletion*	Hemi	X91^0^	Alive	(13, 33)
P150	EB	MEX	M	*CYBB*	del. ex. 1_13	del. ex. 1_13 + McLeod	−	Large deletion (≥1 ex.)	Hemi	X91^0^ + MacLeod	Alive	(13, 33)
P151	DZsi	MEX	M	*CYBB*	del. ex. 1_13	del. ex. 1_13	−	Large deletion (≥1 ex.)	Hemi	X91^0^	Alive	(13)
P152	EC	MEX	M	*CYBB*	ex. 5	c.345C>G	p.His115Gln	*Missense*	Hemi	X91^−^	Alive	(13, 29)
P153	ED	MEX	M	*CYBB*	del. ex. 1_13	del. ex. 1_13	−	Large deletion (≥1 ex.)	Hemi	X91^0^	Dead	(13)
P154	EEsi	MEX	M	*CYBB*	ex. 13	c.1612G>T	p.Gly538*	*Nonsense*	Hemi	X91^0^	Dead	(13)
P155	EEsi	MEX	M	*CYBB*	ex. 13	c.1612G>T	p.Gly538*	*Nonsense*	Hemi	X91^0^	Alive	(13)
P156	EF	MEX	M	*CYBB*	ex. 13	c.1678G>T	p.Gly560*	*Nonsense*	Hemi	X91^?^	Alive	(3)
P157	EG	MEX	M	*NCF1*	ex. 2	c.75_76delGT	p.Tyr26Hisfs*26	*Deletion*	Homo	A47^0^	Dead	(4)
P158	EH	MEX	M	*CYBB*	ex. 5	c.374G>A	p.Trp125*	*Nonsense*	Hemi	X91^?^	Dead	(3)
P159	EI	MEX	M	*NCF1*	ex. 2	c.75_76delGT	p.Tyr26Hisfs*26	*Deletion*	Homo	A47^0^	Alive	(4)
P160	EJ	MEX	M	*CYBB*	ex. 9	c.1085C>G	p.Thr362Arg	*Missense*	Hemi	X91^?^	Alive	(3)
P161	EK	MEX	M	*CYBB*	ex. 3	c.142-1G>A	p.Ser48_Ala84del (del. ex. 3?)	*Splicing*	Hemi	X91^?^	Dead	(3)
P162	EL	MEX	F	*NCF2*	ex. 2/ex. 5	c.55_63del/c.661C>T	p.Lys19_Asp21del/p.Gln221*	*Deletion*/*Nonsense*	Comp	A67^0^/A67^?^	Alive	(4, 13)
P163	EM	MEX	M	*NCF2*	ex. 2	c.55_63del	p.Lys19_Asp21del	*Deletion*	Homo	A67^0^	Dead	(4)
P164	EN	MEX	M	*CYBB*	ex. 9	c.1148C>T	p.Pro383Leu	*Missense*	Hemi	X91^?^	Alive	(16)
P165	EO	MEX	M	*CYBB*	ex. 5	c.388C>T	p.Arg130*	*Nonsense*	Hemi	X91^0^	Alive	(3, 29, 31)
P166	EP	MEX	M	*CYBB*	ex. 6	c.626A>G	p.His209Arg	*Missense*	Hemi	X91^0^	Dead	(3, 13, 29)
P167	EQ	MEX	M	*NCF2*	ex. 2	c.175delG	p.Ala59Profs*40	*Deletion*	Homo	A67^?^	Alive	ND
P169	ES	MEX	M	*CYBB*	ex. 12	c.1473del	p.Phe491Leufs*11	*Deletion*	Hemi	X91^?^	Dead	(13)
P170	DXso	MEX	M	*CYBB*	ex. 7	c.676C>T	p.Arg226*	*Nonsense*	Hemi	X91^0^	Dead	(3, 13, 29)
P171	ET	MEX	M	*CYBB*	ex. 6	−	p.Trp206*	*Nonsense*	Hemi	X91^0^	Dead	(13)
P172	EU	MEX	M	*CYBB*	ex. 7	c.742dupA	p.Ile248Asnfs*36	*Insertion*	Hemi	X91^0^	Alive	(13)
P173	EV	MEX	M	*CYBB*	ex. 2	c.141+1G>T	del. ex. 2? (p.Leu16_Gly47del)	*Splicing*	Hemi	X91^−^	Alive	(3, 13, 29)
P174	EW	MEX	M	*CYBB*	ex. 7	c.752G>A	p.Trp251*	*Nonsense*	Hemi	X91^0^	Alive	(3, 29)
P175	EX	MEX	F	*NCF2*	ex. 2/ex. 2	c.55_63del/c.74C>A	p.Lys19_Asp21del/p.Ala25Asp	*Deletion*/*Missense*	Comp	A67^0^/A67^?^	Alive	(4, 13)
P176	EY	MEX	M	*CYBB*	del. ex. 1_13	del. ex. 1_13	−	Large deletion (≥1 ex.)	Hemi	X91^0^	Alive	(13)
P177	EZ	MEX	M	*CYBB*	ex. 10	c.1275C>A	p.Tyr425*	*Nonsense*	Hemi	X91^?^	Alive	(3, 29)
P178	FA	MEX	M	*CYBA*	ex. 5	c.354C>A	p.Ser118Arg	*Missense*	Homo	A22^0^	Alive	(4)
P179	FBsi	MEX	M	*CYBB*	ex. 6	c.580del	p.Thr194Profs*20	*Deletion*	Hemi	X91^0^	Alive	(13, 33)
P180	FC	MEX	M	*NCF1*	ex. 2	c.75_76delGT	p.Tyr26Hisfs*26	*Deletion*	Homo	A47^0^	Alive	(4)
P181	FD	MEX	M	*CYBB*	ex. 10	−	p.GIy412Val	*Missense*	Hemi	X91^−^	Dead	(13)
P184	FG	MEX	M	*CYBB*	ex. 7	c.676C>T	p.Arg226*	*Nonsense*	Hemi	X91^0^	Dead	(3, 13, 29)
P185	CPc	MEX	M	*CYBA*	ex. 1	c.4_24del21	p.Gly2_Met8del	*Deletion*	Homo	A22^0^	Alive	(13)
P186	FH	MEX	F	*NCF2*	ex. 2	c.229C>T	p.Arg77*	*Nonsense*	Homo	A67^0^	Alive	(4)
P187	FIc	MEX	M	*CYBB*	ex. 1	c.13delG	p.Ala5Leufs*2	*Deletion*	Hemi	X91^−^	Alive	(13)
P188	FIc	MEX	M	*CYBB*	ex. 1	c.13delG	p.Ala5Leufs*2	*Deletion*	Hemi	X91^−^	Dead	(13)
P189	FJ	MEX	M	*CYBB*	del. ex. 1_3	del. ex. 1_3	−	Large deletion (≥1 ex.)	Hemi	X91^0^	Alive	(3, 13)
P190	FK	MEX	M	*CYBB*	ex. 2	c.80_83delTCTG	p.Val27Glyfs*33	*Deletion*	Hemi	X91^0^	Alive	(3, 13, 33)
P192	FM	MEX	M	*NCF2*	in. 3/ex. 1	c.366+1G>A/c.124A>C	del. ex. 3 e 4/p.Asn42His	*Splicing/Missense*	Comp	A67^0^/A67^?^	Alive	(4)/ND
P193	DGn	MEX	M	*CYBB*	ex. 9	c.978delT	p.Phe326Leufs*17	*Deletion*	Hemi	X91^?^	Dead	ND
P194	FN	MEX	F	*NCF2*	ex. 1	c.137T>G	p.Met46Arg	*Missense*	Homo	A67^0^	Alive	(13, 31)
P196	FP	MEX	M	*CYBB*	ex. 3	c.207delinsTT	−	*Indel*	Hemi	X91^0^	Alive	(13, 31)
P197	FQ	MEX	M	*CYBB*	del. ex. 1_3	del. ex. 1_3	−	Large deletion (≥1 ex.)	Hemi	X91^0^	Alive	(3, 13)
P198	EJ	MEX	M	*CYBB*	ex. 9	c.1085C>G	p.Thr362Arg	*Missense*	Hemi	X91^?^	Alive	(3)
P199	FR	MEX	M	*CYBB*	in. 8	c.898-1G>A	del. ex. 9? (p.Val300_Pro383del)	*Splicing*	Hemi	X91^0^	Dead	(13, 31)
P200	FS	MEX	M	*CYBB*	del. ex. 3	c.252G>A	del. ex. 3 (p.Ser48_Ala84del)	*Splicing*	Hemi	X91^0^	Alive	(3, 29)
P201	CSu	MEX	M	*CYBB*	ex. 9	c.1016C>A	p.Pro339His	*Missense*	Hemi	X91^0^	Alive	(3, 13, 29)
P203	FU	MEX	M	*CYBB*	ex. 12	c.1521_1523del	p.Lys508del	*Missense*	Hemi	X91^?^	Alive	(3, 13)
P204	FV	MEX	M	*CYBB*	ex. 12	c.1508C>A	p.Thr503Lys	*Missense*	Hemi	X91^+^	Alive	(13)
P205	FW	MEX	F	*CYBB*	ex. 7	c.676C>T	p.Arg226*	*Nonsense*	Hetero	X91^0^ #	Dead	(3,29)
P206	FX	MEX	M	*CYBB*	Prom	c.-65C>T	−	*Deletion*	Hemi	X91^0^	Alive	(3, 13, 29)
P207	FY	MEX	M	*CYBB*	ex. 9	c.1038delT	p.Glu347Argfs*39	*Deletion*	Hemi	X91^0^	Dead	(3, 13, 29)
P208	FZ	MEX	M	*CYBB*	ex. 10	c.1234G>A	p.Gly412Arg	*Missense*	Hemi	X91^−^	Dead	(3, 13)
P209	GA	MEX	M	*CYBA*	ex. 4	c.287T>C	p.Leu96Pro	*Missense*	Homo	A22^0^	Alive	(13)
P210	FBsi	MEX	M	*CYBB*	ex. 6	c.580del	p.Thr194Profs*20	*Deletion*	Hemi	X91^0^	Dead	(13, 33)
P214	GD	MEX	M	*CYBB*	ex. 7	c.676C>T	p.Arg226*	*Nonsense*	Hemi	X91^0^	Alive	(3, 13, 29)
P215	GE	CHI	F	*NCF2*	ex. 8–9	Duplication	−	Duplication	Homo	A67^?^	Dead	ND
P216	GF	CHI	M	*CYBB*	ex. 7	c.676C>T	p.Arg226*	*Nonsense*	Hemi	X91^0^	Alive	(3, 13, 29)
P217	GG	CHI	M	*CYBB*	ex. 7	c.781C>T	p.Gln261*	*Nonsense*	Hemi	X91^0^	Alive	(3, 29)
P219	GI	CHI	M	*CYBB*	in. 6	c.675-2A>G	Splicing site	*Splicing*	Hemi	X91^?^	Alive	ND
P223	GM	CRC	F	*NCF1*	ex. 2	c.75_76delGT	p.Tyr26Hisfs*26	*Deletion*	Homo	A47^0^	Alive	(4)
P224	GN	CRC	M	*CYBB*	ex. 7	c.725_726delCA	p.Thr242Serfs*2	*Deletion*	Hemi	X91^?^	Alive	(31)
P225	GO	CRC	M	*CYBB*	ex. 1	c.34delA	p.Ile12Phefs*10	*Deletion*	Hemi	X91^?^	Alive	ND
P226	GP	ARG	M	*CYBB*	ex. 9	c.1096T>G	p.Trp361Gly	*Missense*	Hemi	X91^?^	Alive	ND
P227	GQc	ARG	M	*CYBB*	ex. 13	c.1598_1600delGAG	p.Gly533del	*Deletion*	Hemi	X91^−^	Alive	(3)
P228	GQc	ARG	M	*CYBB*	ex. 13	c.1598_1600delGAG	p.Gly533del	*Deletion*	Hemi	X91^−^	Dead	(3)
P229	GR	ARG	M	*CYBB*	ex. 2	Del. ex. 2	del. ex. 2 (p.Leu16_Gly47del)	*Splicing*	Hemi	X91^0^	Alive	(3)
P234	GW	PER	M	*CYBB*	ex. 9	c.1081T>C	p.Trp361Arg	*Missense*	Hemi	X91^0^	Alive	(3)
P235	GX	PER	M	*CYBB*	ex. 5	c.388C>T	p.Arg130*	*Nonsense*	Hemi	X91^0^	Alive	(3, 29)
P236	GY	PER	M	*CYBB*	in. 9	c.1152-1G>A	Splicing site	*Splicing*	Hemi	X91^0^	Dead	(3)
P237	GZ	URU	M	*CYBB*	in. 3	c.253-8A>G	Splicing site	*Splicing*	Hemi	X91^0^	Alive	(3, 29)

Fam., family; Zygo, zygosity; Hemi, hemizygosity; Homo, homozygosity; Comp., compound heterozygosity; ex., exon; in., intron; Express., expression; Del., deletion; Prom., promoter; ND, not described; BRA, Brazil; MEX, Mexico; CHI, Chile; CRC, Costa Rica; ARG, Argentina; PER, Peru; URU, Uruguay; M, male; F, female. The lowercase letter in the family coding represents the degree of kindred: c, cousin; si, siblings; u, uncle; n, niece/nephew; so, son; m, mother. In the “Type” column, the letter X corresponds to XL inheritance, the letter A corresponds to AR forms, and the number corresponds to the affected protein. Superscript information corresponds to the final effect on the expression or function of the protein: (−) lower expression protein/function; (+) normal protein expression, but with impaired function; (“0”) absence of expression and/or function; (?) lack of information regarding the expression and/or function of the affected protein. #, patients with possible distorted X chromosome inactivation. McLeod phenotype with a large deletion on the X chromosome (XL) that involves some genes such as *CYBB* and *XK* genes (encodes Kx antigen). Nomenclature of mutations according to the recommendations of the American College of Medical Genetics and Genomics and the Human Genome Variation Society (35).

**Figure 2.**
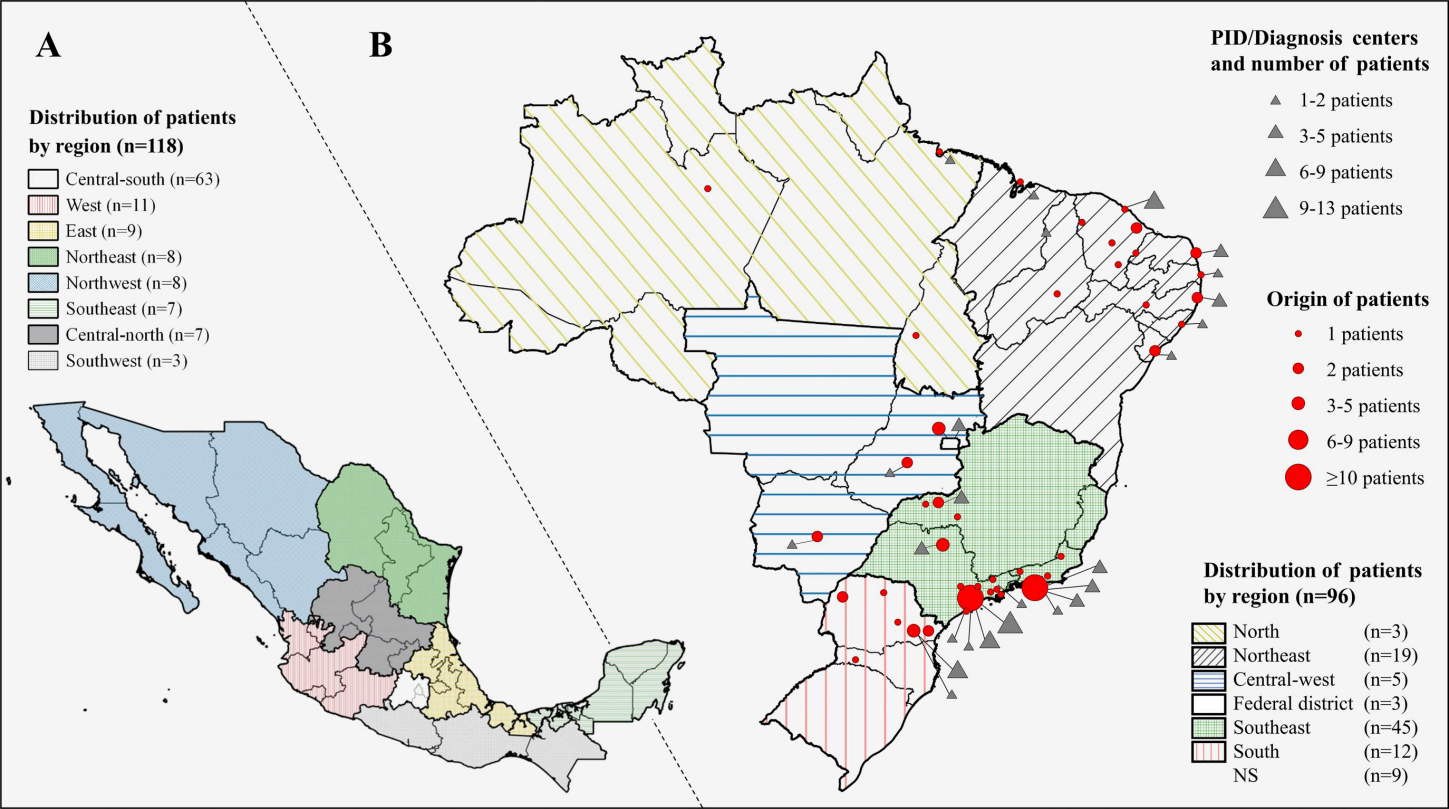
**Geographic distribution of patients with CGD in Brazil and Mexico, and diagnostic/follow-up centers in Brazil. (A)** Distribution of patients with CGD in Mexico by geographic region. **(B)** Geographic distribution of patients with CGD in Brazil and PID diagnosis/follow-up centers by region, represented by classes (circles and triangles, respectively). NS, region of origin not specified.

Brazil and Mexico account for 90% of the patients described here, and the regional distribution of the patients is broad, unlike in other countries, where patients are concentrated in more developed regions. In Mexico, the regional origins were as follows: Northwest (*n* = 8), Northeast (*n* = 8), West (*n* = 11), East (*n* = 9), Central-North (*n* = 7), Central-South (*n* = 63), Southwest (*n* = 3), Southeast (*n* = 7), and unknown (*n* = 2), while in Brazil, the distribution was as follows: North (*n* = 3), Northeast (*n* = 19), Central-West (*n* = 5), Federal District (*n* = 3), Southeast (*n* = 45), South (*n* = 12), and unknown (*n* = 9). The regions with the most patients are also where the main medical and research centers for IEI are located. In Mexico (Central-South, *n* = 63), this is the National Institute of Pediatrics. In contrast, in Brazil (Southeast, *n* = 45), it is the Federal Universities of São Paulo, Rio de Janeiro, and the University of São Paulo (USP) (Fig. 2).

### Analysis of genetic variants

**Figure 3.**
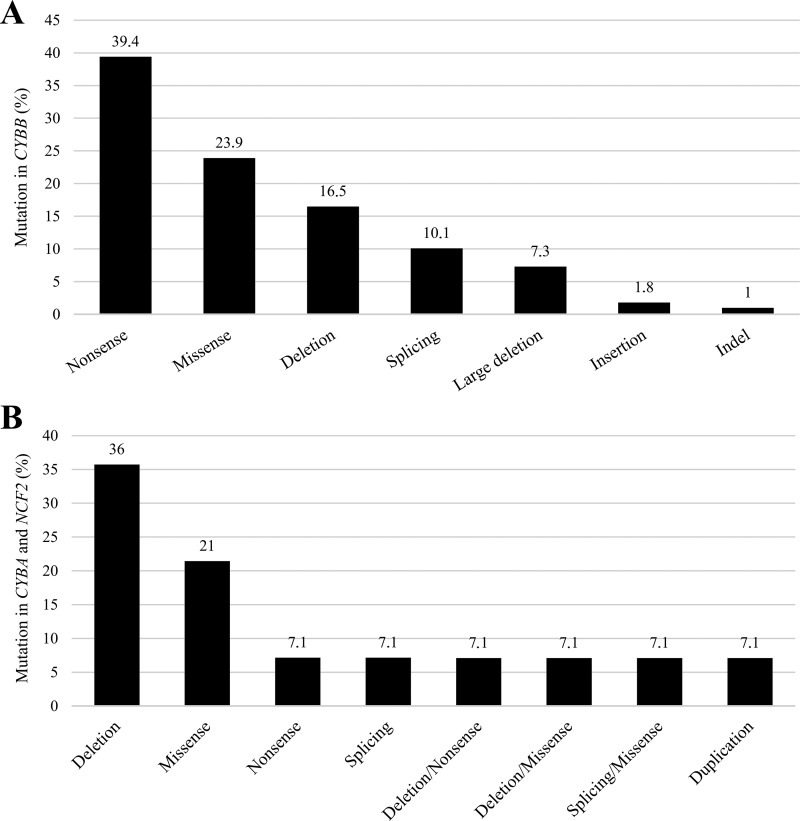
**Heterogeneity of pathogenic variants in the *CYBB*, *CYBA*, and *NCF2* genes. (A and B)** (A) Heterogeneity of mutations in the *CYBB* gene among 109 patients from 94 families and (B) heterogeneity of mutations in the *CYBA* and *NCF2* genes among 14 patients from 13 families.

The genetic diagnosis was obtained for 141 patients (59%) from 122 families, with the majority having XL-CGD (*n* = 109, 77%). 11 novel pathogenic variants were identified, including nine in the *CYBB* gene and two in *NCF2*, all predicted to be deleterious according to Combined Annotation Dependent Depletion (CADD), SIFT, MutationTaster, and POLYPHEN-2 (Table 1). Patients from Mexico comprised 64% of those with genetic analysis (*n* = 91, 77% of all Mexican patients), while Brazil had only 35 patients genetically diagnosed (36.5%). Argentina, Peru, Chile, Costa Rica, Uruguay, and Paraguay had, respectively, four (100%), three (100%), six (67%), five (60%), one (50%), and no patients genetically diagnosed. For several reasons, including lack of genetic material or technical issues, genetic diagnosis was not possible for 97 patients from 88 families. Overall, 109 cases (77%) had XL-CGD, and 32 (22%) had AR-CGD, with 92 different mutations identified. No gene *hot spots* were found, with exonic regions most frequently affected (*n* = 122), followed by splicing sites (intron and exon) (*n* = 13), large deletions (≥1 exon) (*n* = 8), and a single promoter deletion case with no gp91^phox^ expression (P206) (Table 2 and Table S1). Pathogenic variants in *CYBB*, *CYBA*, and *NCF2* were heterogeneous (Fig. 3). Nonsense mutations (*n* = 43) were most frequent in *CYBB*, followed by missense mutations (*n* = 26), deletions (*n* = 18), splice sites (*n* = 11), copy number variation with large deletions covering the *CYBB* region (≥1 exon) (*n* = 8), insertions, and indels (*n* = 3). Patient P150 was the only one with McLeod syndrome.

In *CYBA* and *NCF2*, deletions were most common (*n* = 6), followed by missense (*n* = 3), nonsense, splicing sites, deletion/nonsense, deletion/missense, and splicing site/missense (each in 1 patient). There were four compound heterozygous cases: three in *CYBA* (P162, P175, and P192) and one in *NCF2* (P60) (Fig. 3). All 18 patients with p47^phox^ deficiency from 15 families had the same homozygous mutation, ΔGT (c.75_76delGT), in *NCF1*. No pathogenic variants in *NCF4* (not typical CGD), *CYBC1*, or *RAC* were identified in this study. Data on neutrophil NADPH oxidase expression were obtained through mutation databases, scientific articles, or *in silico* analysis: 63 patients were X91^0^ (undetectable), 9 were X91^−^ (low), three were X91^+^, and 28 were X91^?^ (the level of protein expression was not determined). Among the X91^0^ patients, one had a *de novo* mutation (P146), and one had McLeod syndrome (P150). Two symptomatic carrier mothers (P145 and P205), unrelated, had the same pathogenic variant in exon 7 of *CYBB*, c.676C>T, leading to abolished gp91^phox^ expression—likely cases of skewed X-inactivation (Table 2 and Table S1). Among the AR-CGD cases, 27 patients were homozygous: five were A22^0^, three were A67^0^, 18 were A47^0^, and one was A67^?^ (P167). In cases of compound heterozygosity, mutations c.55_63delAAGAAGGAC (P162 and P175) and c.366+1G>A (P192) led to an absence of p67^phox^ (A67^0^), though the respective allele mutations could not determine protein expression (A67^?^). For compound heterozygous *CYBA* (P60: c.472_484del/c.399delC), protein expression could not be determined (A22^?^) (Table 2 and Table S1).

### Initial clinical manifestations

**Figure 4.**
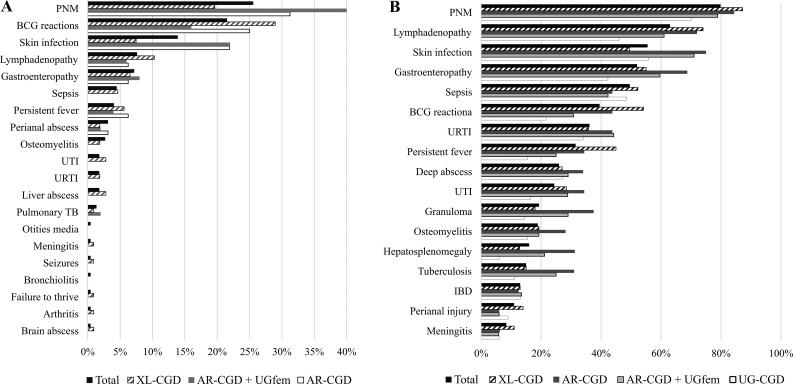
**Relative frequency of first manifestation and clinical manifestations in patients with CGD from Latin America during the follow-up time. (A and B)** (A) First clinical manifestation (*n* = 223) and (B) clinical manifestation during life (*n* = 238). PNM, PNM and other lung infections; hepatosplenomegaly, hepatosplenomegaly, splenomegaly, and hepatomegaly, including liver abscess).

The first clinical manifestation of CGD occurred within the first 2 years of life in 83% (*n* = 188) of our patients, with some cases presenting multiple simultaneous manifestations. Infectious manifestations were predominant, affecting 196 patients (88%), followed by gastroenteropathy, typically expressed as diarrhea (*n* = 16, 7%), with no clear distinction between infectious or inflammatory causes, nonspecific persistent fever (*n* = 9), seizures (*n* = 1), and failure to thrive (*n* = 1). The relative frequency of the first clinical manifestations, categorized by genotype/phenotype, can be seen in Fig. 4 A. PNM was the most common initial clinical manifestation (*n* = 57), followed by adverse reactions to the BCG vaccine (*n* = 48), skin infections (*n* = 32), lymphadenopathy (*n* = 17), gastroenteropathy (*n* = 16), and sepsis (*n* = 9). Analyzing infections by anatomical site, we observed that the lungs (27%) were the most frequently affected, followed by the skin (17%), lymph nodes (8%), and intestines (7%). The sites of BCG infection (21%) were multiple and are described in detail in the section “BCG infectious disease and TB in CGD patients” and in Fig. 6. Although there was no statistical difference, PNM was more frequent among patients with XL-CGD, as were liver abscesses. Conversely, skin infections were more frequent in patients with AR-CGD and in the AR-CGD + UGfem group, manifesting as pyoderma, pustulosis, impetigo, dermatitis, cellulitis, omphalitis, furunculosis, and other abscesses.

### Clinical manifestations and complications in CGD patients

**Figure 5.**
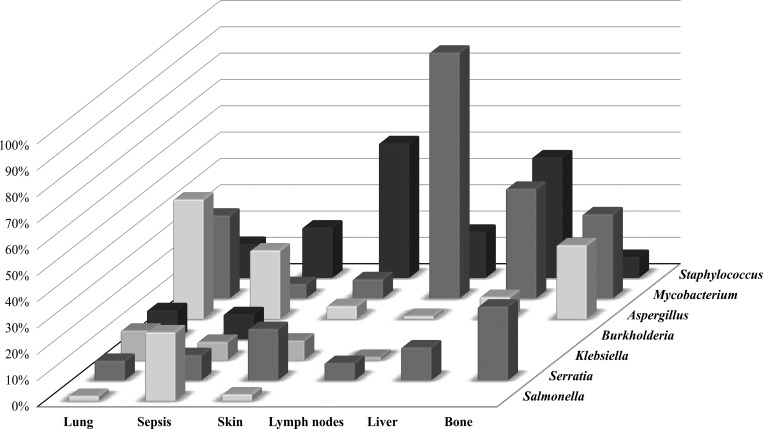
Frequency (%) of microorganisms isolated according to site of infection.

Infectious manifestations occurred in all patients, with PNM being the most frequent, affecting 80% of patients (*n* = 191), with an average of 3.8 episodes per patient (range, 1–25 episodes), showing no significant difference between groups (XL, AR, UG, and AR + UGfem), as shown in Fig. 4 B. Recurrent upper respiratory tract infections (URTIs) were less common than PNM (*n* = 86, 36.1%), with an average of nearly four episodes per patient (range, 1–20). Otitis media was the most common (*n* = 32), followed by tonsillitis/pharyngitis (*n* = 24) and sinusitis (*n* = 12). Among the pathogens identified in patients with PNM, the *M. tuberculosis* complex was the most frequent (17%): *Mycobacterium* spp. (*n* = 14), *M. tuberculosis* (*n* = 8), and *Mycobacterium bovis/BCG* (*n* = 11), followed by *Klebsiella pneumoniae* (*n* = 8), *Burkholderia cepacia* (*n* = 8), *Serratia marcescens* (*n* = 7), *S. aureus* (*n* = 6), and *Pseudomonas aeruginosa* (*n* = 5), with others identified in one to four patients. Regarding fungal infections, *Aspergillus* species were predominant, confirmed in 47 patients (25% of patients with PNM and 76% of patients with fungal PNM); however, species identification, such as *Aspergillus fumigatus*, was made in only five patients. Fig. 5 describes the main microorganisms isolated by infection site. In URTIs, *Streptococcus pneumoniae* and *Streptococcus milleri* were isolated from one patient each, as well as *Staphylococcus* spp., *S. aureus*, and *Pseudomonas fluorescens*. Among fungi, *A. fumigatus*, *Candida* spp., and *Pneumocystis jirovecii* were isolated from one patient each.

The second most frequent clinical manifestation was lymphadenopathy, affecting 63% of patients (*n* = 149), with an average of 3.3 episodes per patient (range, 1–16 episodes). The genus *Staphylococcus* was the most common (16.5%), except in patients with lymphadenopathy due to adverse reactions to the BCG vaccine (*n* = 42, 46% of bacterial lymphadenitis and 93% of patients with lymphadenopathy caused by mycobacteria) (Fig. 4 B and Fig. 5). Skin infections occurred in 132 patients (55.5%), with an average of five episodes per patient (range, 1–37 episodes), with subcutaneous abscesses being the most common manifestation (50%), primarily due to *S. aureus* (*n* = 16, 12%). Patients with AR-CGD and the AR + UGfem group experienced more skin infections than those with XL-CGD (P = 0.032/P = 0.01), although the infections were more frequent in the XL group with 233 episodes compared to 158 in the AR form. Other manifestations occurred in 2.3–8.3% of patients, including pyoderma (caused by *S. aureus* and *K. pneumoniae*), furunculosis (caused by *Staphylococcus* spp. and *K. pneumoniae*), fistulas, cellulitis (caused by *Staphylococcus* spp., *S. marcescens*, and *Chromobacterium violaceum*), and impetigo (caused by *Staphylococcus* spp., *Serratia* spp., and *S. aureus*) (Fig. 4 B and Fig. 5).

One hundred and twenty-three patients (52%) had gastroenteropathy, with an average of 5 episodes per patient (range, 1–25 episodes). Most cases were confirmed or inferred to be infectious; however, only 28% of patients (*n* = 35) had confirmation of the etiological agent in at least one of the episodes. Of these, the majority were bacterial (80%), with the genus *Salmonella* being the most common (*n* = 16, 13%), affecting 46% of patients with isolated microorganisms. Chronic diarrhea (lasting >4 wk) was present in 31 patients, followed by hematochezia (*n* = 8), stomatitis/esophagitis/duodenitis/appendicitis (*n* = 7), and intestinal obstruction due to granulomatous inflammation (*n* = 3). Other events were associated with allergies (*n* = 3) or food intolerance (*n* = 1). Inflammatory bowel disease (IBD) was diagnosed in 31 patients (13%). It was more common among Brazilians (*n* = 21), especially those with XL-CGD (*n* = 7), accounting for 29% of XL-CGD patients in the country (Fig. 4 B and Fig. 5). Perianal lesions were identified in 26 patients (11%), with an average of 3.1 episodes per patient (range, 1–24), the most frequent being perianal abscesses (*n* = 25), followed by fistulas (*n* = 3) and granulomas (*n* = 1). Only four patients (15%) had isolated microorganisms, all bacterial: *K. pneumoniae* (*n* = 2), *Citrobacter freundii* (*n* = 1), and *S. aureus* (*n* = 1). Nearly half of the patients experienced sepsis (*n* = 119), with an average of 1.5 episodes per patient (range, 1–8 episodes), being more recurrent in patients with XL-CGD, 1.6 times more than those with AR-CGD or the AR + UGfem group (P < 0.02). *Salmonella* (*n* = 18) was the most frequent pathogen associated with sepsis, followed by *Staphylococcus* spp. (*n* = 14), primarily *S. aureus* (*n* = 10), *Serratia* spp. (*n* = 7), *Klebsiella* spp. (*n* = 5), and *Burkholderia* spp. (*n* = 6). *Aspergillus* spp. was the principal fungus associated with sepsis (*n* = 16), followed by *Candida* spp. (*n* = 6), and one case associated with *Mucor* spp. infection (Fig. 5). Septic shock was the leading cause of death in this study (*n* = 36), accounting for 45.5% of total deaths.

Deep abscesses occurred in at least five different organs, affecting 61 patients (26%), with an average of 1.7 episodes per patient (range: 1–8 episodes). Hepatic abscesses were the most frequent (*n* = 45, 74%), with two patients experiencing recurrent hepatic abscesses (P124, *n* = 6 and P196, *n* = 8), followed by pulmonary abscesses (*n* = 11), cerebral abscesses (*n* = 10), splenic abscesses (*n* = 3), and intestinal abscesses (*n* = 2). *S. aureus* (*n* = 11) and *M. bovis* (*n* = 9) were the primary etiological agents, followed by *S. marcescens* (*n* = 3) and *Aspergillus* spp. (*n* = 3). Hepatosplenomegaly was clinically diagnosed in 30 patients (16%), with an average of 1.5 episodes per patient (range: 1–9 episodes), and was recurrent in only two patients (P63, *n* = 9 and P196, *n* = 8). Eight patients had only hepatomegaly (one episode each), and three had only splenomegaly. 10 patients had hepatosplenomegaly concomitant with a hepatic abscess, one with a splenic abscess (P192), and one with both hepatic and splenic abscesses (P196). Urinary tract infections (UTIs) occurred in 58 patients (24%), with an average of two episodes per patient (range, 1–12 episodes), and were more frequent in non-Brazilian patients (P = 0.049), particularly among Mexican patients (P = 0.008). *K. pneumoniae* and *Klebsiella oxytoca* (*n* = 5), *Escherichia coli* (*n* = 4), and *Salmonella enterica* (*n* = 2) were the main microorganisms isolated.

Osteomyelitis was diagnosed in 45 patients (19%), with an average of 1.3 episodes per patient (range, 1–4 episodes), and was more frequent in non-Brazilian patients (P = 0.038). *Serratia* spp. (*n* = 7), *M. bovis* (BCG dissemination), *Aspergillus* spp. (*n* = 4), and *A. fumigatus* (*n* = 3) were the most common, with only one case of osteomyelitis caused by *Scedosporium* spp. (P227) (Fig. 5). Infections of the meninges affected 20 patients (8.4%), with an average of 1.25 episodes per patient (range, 1–2 episodes). *Mycobacterium* spp. were the most common (*n* = 6), including at least two cases of TB, including one case of neurotuberculosis (P11). Granulomas were identified in 46 patients (19.3%), with an average of 3 episodes per patient (range 1–12). Granulomas were most commonly found in lymph nodes (43.5%), lungs (34.8%), skin (21.7%), and intestines/mesentery (13%). Despite the low isolation rate, the genus *Mycobacterium* was the most frequently isolated group (*n* = 8, 17% of the total), with *M. bovis* identified in five of these cases (reaction to BCG). The genus *Aspergillus* caused pulmonary granulomas in two patients (P33 and P136).

### BCG infectious disease and TB in CGD patients

**Figure 6.**
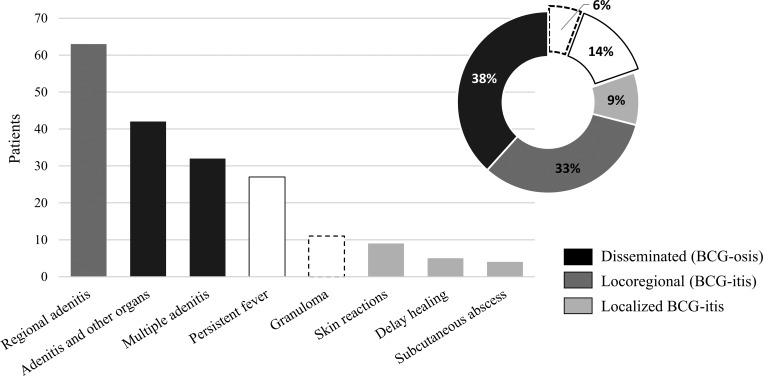
**Classification of the adverse reactions manifestations to the BCG vaccine (*n* = 94).** All 62 patients with locoregional reactions are in the first column (dark gray on the left). The patients (*n* = 48) who had disseminated BCG infection (BCG-osis) were divided into two groups: adenitis (lymphadenopathy) and other organs and multiple adenitis. The patients (*n* = 15) with only local reactions are described in the light gray bars on the right. Granuloma and persistent fever were present in the three groups of reactions to the BCG vaccine. The pie chart presents BCG-osis (black), locoregional BCG-itis (dark gray), localized BCG-itis (gray), persistent fever (white), and granuloma (dashed line) as a percentage of affected patients.

In all participating countries, the BCG vaccine is mandatory at birth (36). 208 patients (87%) were vaccinated with BCG. Among the 30 patients without vaccination confirmation, six did not receive the vaccine due to a family history of CGD, and P156 did not receive the vaccine as it was not administered in the United States of America (USA) where they were born. 94 patients (45%) experienced an adverse reaction, with an average age of 5 mo (range, 0.25–51 mo), and this was the first infectious manifestation of CGD in 48 individuals (51%). 15 patients (16%) had a local reaction (fistulization at the vaccination site, subcutaneous abscess, or delayed healing), and 62 (66%) had a locoregional reaction (regional adenitis), both forms of BCG-itis. 48 patients (51%) had disseminated BCG infection (BCG-osis) (Fig. 6). Pharmacological treatment was administered to 73 patients (77.6%) with BCG-itis, including rifampicin, isoniazid, ethambutol, and pyrazinamide, as well as surgical resection of axillary/cervical lymph nodes in some cases. Although pyrazinamide is ineffective against BCG strains due to intrinsic resistance, it was initially included in empirical TB treatment in a few patients before the diagnosis of BCG infection was established. In all such cases, pyrazinamide was discontinued once BCG was confirmed. The average age of presentation of BCG-osis was 5.2 mo (range: 0.25–36 mo) and affected various anatomical sites: systemic lymph nodes (*n* = 36); liver (*n* = 14); lungs (*n* = 13); bone (*n* = 7); spleen (*n* = 6); regional lymph node and lung (*n* = 4); regional lymph node, lung, liver, and bone (*n* = 2); intestine (*n* = 2); regional lymph node, lung, and bone (*n* = 1); and regional lymph node, lung, liver, and spleen (*n* = 1) (Fig. 6). Anti-TB treatment was administered to 42 patients with BCG-osis (87.5%), with the combination of rifampicin and isoniazid being the most commonly used (*n* = 38). Three of the seven patients with BCG-osis who did not receive anti-TB treatment died shortly after diagnosis, and two due to septic shock (P166 and P181). Generally, adverse reactions to BCG were more frequent in patients with XL-CGD (P = 0.002), as was BCG-itis (P < 0.001), but not BCG-osis, for which no significant association was identified. Less frequent than BCG reactions, TB was diagnosed in 37 patients (15%), with a median age of 1 mo (range: 1–5 mo), and *M. tuberculosis* confirmation in only 35% of cases (*n* = 13). Pulmonary TB was the most common (*n* = 24), followed by disseminated forms affecting lymph nodes (*n* = 10), meninges (*n* = 5), intestine (*n* = 3), bones (*n* = 2), spleen (*n* = 2), skin (*n* = 1), and brain (*n* = 1). BCG infection and TB did not manifest simultaneously, and no cases of environmental mycobacterial infection were confirmed.

### Treatment and hospitalization

Of the total patients, 227 (95%) used cotrimoxazole (sulfamethoxazole [SMX] + trimethoprim [TMP]) for bacterial prophylaxis. One patient, who was intolerant to SMX, substituted it with doxycycline + TMP (P57), while the remaining 10 patients died shortly after diagnosis, which was made in a hospital setting. Regarding antifungal prophylaxis, 207 patients (87%) used azole antifungals, with itraconazole being the most commonly used (97%), and the combination of cotrimoxazole and itraconazole was employed in 187 patients (79%) (37). IFN-γ prophylaxis was adopted by 93 patients (39%) from only three countries (Mexico, Brazil, and Argentina), predominantly in XL-CGD patients (63%) and consistently in conjunction with TMP-SMX, with Mexico being the primary country, having 83 patients (89%). The main reason for discontinuing of IFN-γ prophylaxis was adverse effects, including persistent fever, headache, myalgia, and skin manifestations. Corticosteroids were used by 94 patients (39%) to manage inflammatory processes, ranging from the nasal application of fluticasone for URTI episodes to oral or intravenous administration for more intense inflammation in the intestines (acute or chronic colitis, IBD, and Crohn-like IBD), lungs, granulomas, and hepatic abscesses that were poorly responsive to antibiotics.

Currently the only curative therapy for CGD, HSTC was performed in 53 patients (22%), totaling 68 transplants. Among these, 41 patients underwent a single transplant (60%), and 12 required a second transplant due to primary or secondary graft failure or graft-versus-host disease (GVHD). Of these, three patients needed a third transplant—one for primary failure (P3), another for secondary failure (P149), and the third for reactivation of cytomegalovirus (CMV) (P15). For the first HSCT, bone marrow was the primary stem cell source for 35 patients (66%), followed by peripheral blood (11, 20%) and umbilical cord blood (6, 11%), with data not available for patient P235. The median age at the first HSCT was 4 years (range, 10–236 mo). For the second HSCT, bone marrow was the primary cell source, except for patient P19, who received umbilical cord cells. For the third HSCT, bone marrow (67%) and peripheral blood (33%) were the cell sources. Notably, more Brazilian patients underwent HSCT (32 patients, 60%), with the majority lacking a defined genetic diagnosis (26 patients, 81%). 26 patients with UG-CGD underwent HSCT (49%), 22 with XL-CGD (42%), and only 5 with AR-CGD (9%): 2 patients with p47^phox^ deficiency (P16 and P157) and 3 with p67^phox^ deficiency (P162, P163, and P192). The success rate for the first HSCT was 56%, for the second HSCT was 66.6%, and for the third HSCT was 66.7%, with a total success rate of 70% among all transplants. Post-HSCT reactions were observed in 40 patients (75%) following the first transplant, with 18 achieving successful recovery: 10 cases of GVHD (P82, P83, P119, P148, P151, P156, P162, P204, P216, and P235), 4 cases of CMV reactivation (P17, P38, P87, and P91), 3 cases of secondary graft failure and infection (P81, P155, and P217), and 1 case of primary graft failure (P173). Among those who received a second transplant, four were due to primary graft failure, five were due to secondary failure, one was due to CMV reactivation, and two cases were unspecified. Two cases of primary graft failure (P3 and P149) and one case of CMV reactivation (P15) required a third transplant. Primary graft failure occurred again in patient P3, who deteriorated with worsening pulmonary conditions (*Aspergillus* spp. and *K. pneumoniae* in blood cultures) and died from septic shock.

**Figure 7.**
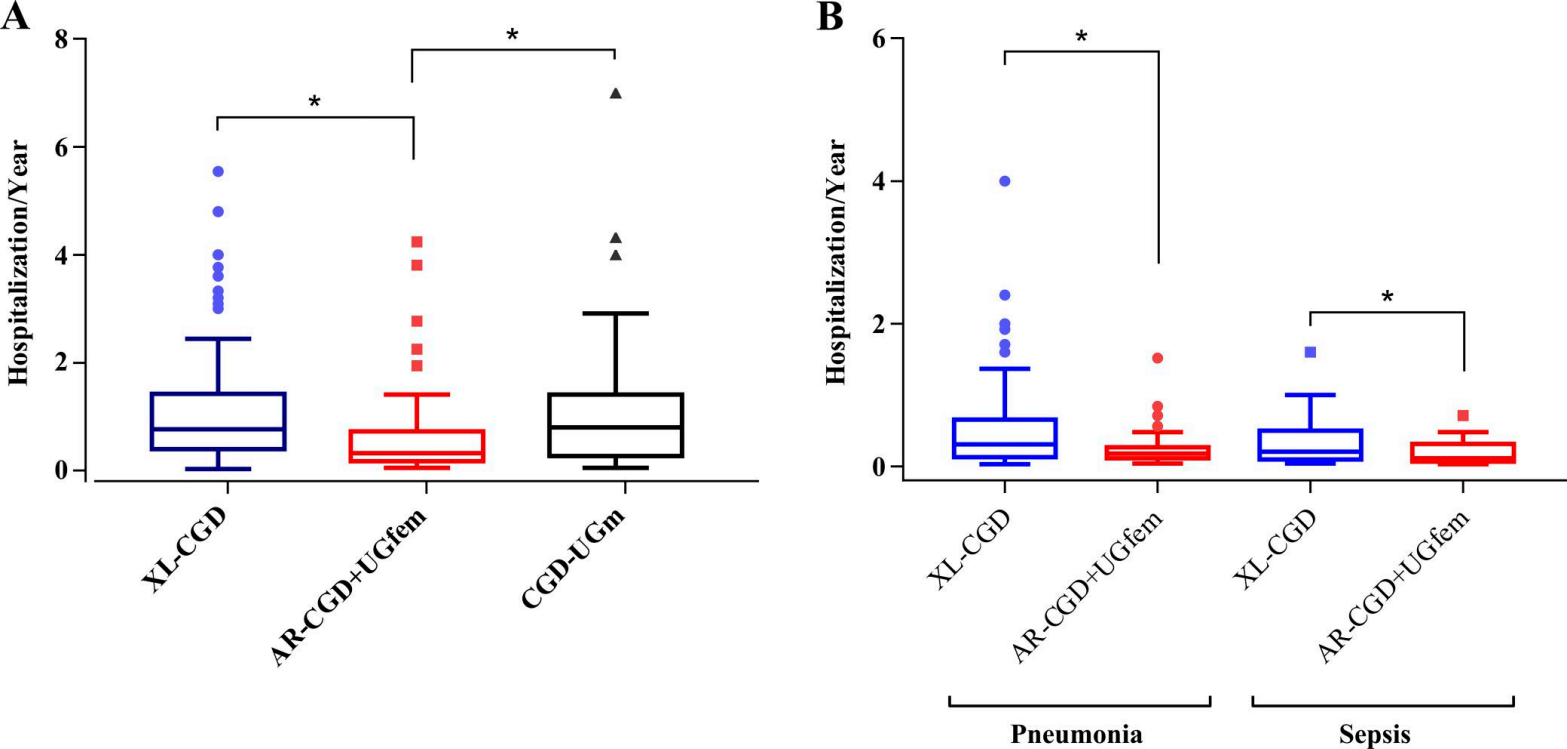
**Number of hospitalizations per year during follow-up of patients with CGD. (A and B)** (A) Hospitalizations of patients with XL-CGD, AR-CGD (AR-CGD + UGfem), and male patients with UG (CGD-UGm), P < 0.05 (Kruskal–Wallis test); (B) hospitalizations due to the main causes of patient death (PNM and sepsis), P < 0.05 (Mann–Whitney test). *Statistical difference.

225 patients (95%) required at least one hospitalization during the follow-up period, with 40 needing intensive care at least once. When evaluating the number of hospitalizations per year (total hospitalizations/follow-up time in years), patients with XL-CGD had a higher frequency compared to those in the AR-CGD + UGfem group (P < 0.05) but not compared to patients in the CGD-UG male (UGm) group (P = 0.4051). The CGD-UGm group, in turn, showed a difference from the AR-CGD + UGfem group (P < 0.05), as shown in Fig. 7 A. PNM and sepsis were the leading causes of hospitalization, as well as the primary causes of death among patients in this study. PNM was the cause of hospitalization for 175 patients (range, 1–12 hospitalizations), while sepsis caused hospitalization in 106 patients (range, 1–8 hospitalizations). Both conditions were more frequent among patients with XL-CGD (P < 0.05), as illustrated in Fig. 7 B.

### Survival and mortality analysis

**Figure 8.**
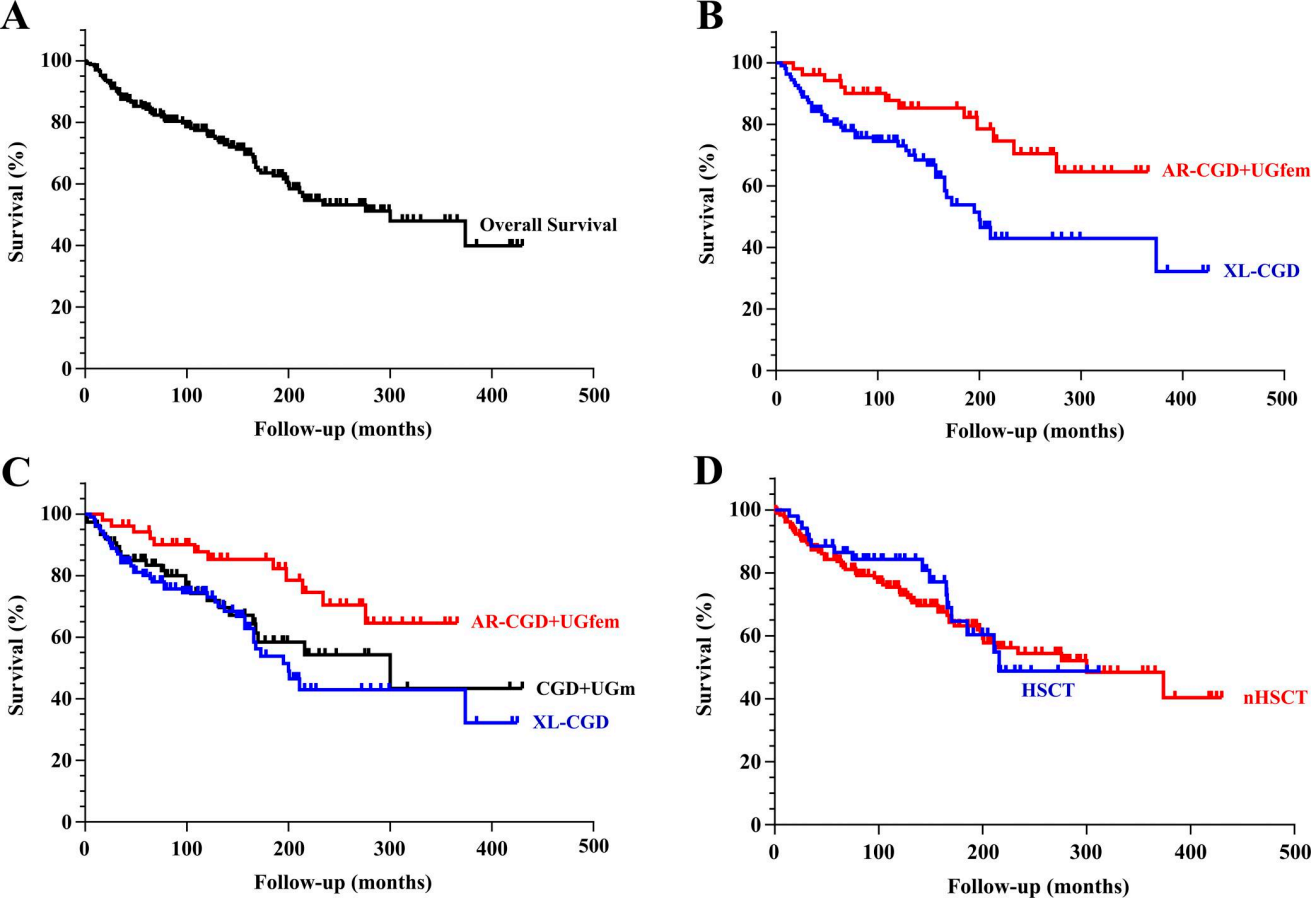
**Kaplan–Meier survival curves. (A–D)** (A) Overall survival (*n* = 236); (B) comparison of overall survival (OS) between XL-CGD (*n* = 108) and AR-CGD + UGfem (*n* = 52), P = 0.004 (log-rank Mantel–Cox); (C) comparison of OS between XL-CGD (*n* = 77), UGm, and AR-CGD + UGfem, P = 0.018; and (D) comparison of OS between transplanted patients (HSCT) and non-transplanted patients (nHSCT), P = 0.689.

80 died (one-third of the total), predominantly male (*n* = 69, 86%). Seven deaths occurred during data collection (six males, including three with XL-CGD and one female with AR-CGD). The overall survival analysis yielded a median of 300 mo (range: 1 mo–35 years), excluding two patients: one due to missing birth date (P83) and the other due to missing date of death (P169). The overall survival rate for the 236 patients (total) was 84.3% at 5 years and 76.1% at 10 years (Fig. 8 A). The median survival for patients with XL-CGD was 200 mo, lower than for patients in the AR-CGD + UGfem group and very similar to the CGD-UGm group, as shown in Fig. 8, B and C (P = 0.0247). Brazilian patients exhibited better overall survival than those from other countries (P = 0.0013). Analysis of overall survival between transplanted (HSCT) and non-transplanted patients did not show a significant difference between groups, as depicted in Fig. 8 D (P = 0.728). The median age at death for patients with CGD was 66 mo (5 years 6 mo; 1 mo–31 years), with XL-CGD at 60 mo (range: 5 mo–31 years), AR-CGD + UGfem at 88 mo (range: 17 mo–23 years), and CGD-UGm at 75 mo (range: 1 mo–25 years). Mortality among patients with XL-CGD was higher (*n* = 43), accounting for 54% of the total deaths, compared to only four deaths in patients with AR-CGD, 12 in the AR-CGD + UGfem group, and 25 deaths in the CGD-UGm group.

Infections were the primary causes of death in patients with CGD, both with and without genetic diagnosis (82.5%), with septic shock (*n* = 36) and PNM (*n* = 21) accounting for a total of 70% of deaths. *Aspergillus* spp. infection was the leading cause of septic death (40% of cases with confirmed etiology), all originating from pulmonary sources. Among the deaths due to PNM, only 13 had isolated microorganisms, including eight cases of *Aspergillus* spp. (one with co-infection of *Aspergillus flavus* and *Aspergillus versicolor*—P109, and another with *A. fumigatus* and *B. cepacia*—P110), four of which involved dissemination of the infection and septicemia, and one case of disseminated aspergillosis after HSCT (P157). *Candida* species were present in five patients: four with septic shock (P129 and P132 with *Candida* spp., P138 with *Candida albicans*, and P121 with co-infection of *Salmonella* sp. and *C. albicans*) and one with PNM associated with *Candida* sp. (P134), although it is not possible to say that there was no cross-contamination by colonization in catheters or probes during hospitalization in at least one or more cases. Deaths due to PNM related to other fungal infections included *Mucor* sp. (P9), *Histoplasma* sp. (P205), and one case with fungal structures in bronchoalveolar lavage (P126). Different bacteria were also isolated from deceased patients: *B. cepacia* (P105, P109, and P139), *S. enterica* (P113 and P171), *Salmonella* sp. and *C. albicans* (P121), *S. marcescens* (P131), *S. aureus* (P231), and *Pseudomonas* spp. and *M. tuberculosis* complex (P11) in cases of septic shock. Other infectious causes of death included phagocyte activation syndrome/hemophagocytic lymphohistiocytosis (P99, P100, P188, and P228), pulmonary TB (P54), infectious myocarditis (P170), severe gastroenteropathy (P193), and two deaths due to BCG dissemination: one from PNM and respiratory failure (P236) and another from septic shock originating from the pulmonary focus (P147), both with XL-CGD, along with one case of disseminated fungal infection after HSCT (P19). Non-infectious causes included autoimmunity: autoimmune thrombocytopenia and hemorrhage in the central nervous system following gene therapy (P158) and lupus nephropathy (P215), neoplasia (craniopharyngioma—P108), and complications after HSCT (GVHD—P59, P86, P89, and P94; thrombotic microangiopathy—P65).

The crude mortality rate did not significantly differ between groups (P = 0.117). However, the specific mortality rate due to PNM among patients with XL-CGD (12.8%) was statistically significant when compared to the AR-CGD + UGfem group (1.9%) (P = 0.038), although no significant difference was observed for septic death (P = 0.272). When examining the case-fatality rate for PNM and sepsis among the groups, a higher case-fatality rate for PNM was noted within the XL-CGD group (14.7%), with 66.7% of deaths due to PNM, compared to the AR-CGD + UGfem group (2.4%), with only 5% of deaths due to PNM (P = 0.035), no significant differences were observed for deaths due to sepsis (P >0.3). PNM was the cause of death exclusively in male patients (66.7% were XL-CGD patients), except for one female carrying a mutation in the *CYBB* gene (P205), considered as having XL-CGD, and another female with a UG (P212). When assessing association measures, the prevalence ratio for patients with XL-CGD dying from PNM is 6.68 times higher than that of patients in the AR-CGD + UGfem group (1.19–39.4–95% confidence interval [CI]), as is the odds ratio, which is 7.52 times higher (1.15–81.2–95% CI), without confirming this association in cases of death due to sepsis.

## Discussion

This study, the largest in Latin America, highlights the genetic and clinical diversity of CGD patients across the region and provides valuable insights into the disease’s epidemiology, treatment outcomes, and challenges. The high rate of XL-CGD observed is consistent with global patterns (8, 10, 11, 12, 13); however, the relatively high frequency of AR forms reflects the social context of certain Latin American regions, where consanguinity is relatively more common, with 46% consanguinity among patients with AR-CGD versus 20% overall. This is considerably lower than the consanguinity rates observed among Arabs and Israeli Jews, where consanguineous marriage is frequent, with high consanguinity rates and AR-CGD cases similar or even higher than XL-CGD (11, 38). The genetic diversity identified, particularly the novel pathogenic variants in *CYBB* and *NCF2*, highlights the necessity for region-specific genetic resources and diagnostic tools to improve early detection and management, especially concerning Brazilian patients.

The results highlight considerable delays in diagnosis, with most diagnoses made more than a year after symptom onset. This delay is likely due to limited awareness, diagnostic resources, and healthcare disparities across Latin America. Our work demonstrated the lowest median/mean age of definitive diagnosis when compared to large-scale studies: USA (64.8 mo) (39), Europe (63.6 mo) (10), Latin American studies, such as in Mexico (30 mo) (13), and the last and largest study on CGD in the region (52.7 mo) (8). The same pattern can be observed when we evaluated patients with XL-CGD and AR-CGD, demonstrating that efforts for early diagnosis in recent years have shown positive results. Early diagnosis is crucial in CGD, as prompt treatment and monitoring can reduce infection-related morbidity and mortality, as well as increase the chances of successful HSCT. Recent results from Europe (the largest series of transplants in patients with CGD reported to date) have shown that even with haploidentical transplants there was a substantial improvement in overall survival and event-free survival in patients with CGD, with even better results with compatible donors and younger patients (24). Among the 53 transplanted patients, 2 did not have age of the first transplant. Of the remaining patients, 10 were transplanted up to 12 mo after diagnosis, and the others after 1 year of definitive diagnosis (76.7%), ranging from 13 mo to 13 years 11 mo. Despite clinical stability at the time of transplantation, many patients exhibited chronic infections, potentially affecting therapeutic outcomes. Since there is insufficient information on the conditioning regimen, it is not possible to discuss the relationship between conditioning and outcome.

All transplants were performed in referral hospitals for pediatrics and HSCT. 34 patients were transplanted when ≤8 years old (64%) and 17 >8 years (32%). When we evaluated the survival of the two groups (eight deaths in each group), we found more favorable survival in patients who underwent HSCT earlier (P = 0.01), as observed in other series (24, 40). Thus, a relative benefit in the success rate of HSCT and survival of Latin American patients may be obtained with the earliest possible transplant, since most transplants in this region are performed when previous therapies or prophylaxis have not demonstrated efficacy in the long term. This suggestion is partly based on the two important declines in the transplant curve observed in Fig. 8 D, referring to Brazilian patients who died a few months after HSCT (P3, P59, P79, and P86—first decline, and P18 and P94—second decline), even with new transplant attempts in some of them (P3 [3×]; P59 and P79 [2×]). Patient P94, a supposed success, presented GVHD that severely affected the lung, leading to acute chronic respiratory failure—PNM with bronchiectasis—dying 5 years after HSCT.

The age of onset of symptoms and the diagnostic delay varies between studies, but the trends are consistent across developing countries (14). The difference in age of diagnosis is due to the considerable variation in manifestations among patients, even in developed countries (9, 10, 16, 41), such as the early age of first clinical manifestations and more severe events in XL-CGD and generally later onset of the disease with less severe and frequent events in AR-CGD. Implementing more widespread newborn screening for CGD in Latin America could mitigate these delays, facilitating timely intervention and improving outcomes.

Infectious complications, particularly with pathogens such as *Aspergillus* and *Staphylococcus*, remain the primary challenge for CGD patients, where invasive *Aspergillus* spp. infections remain a significant challenge despite the implementation of azole antifungals, the main cause of severe pulmonary infections and death in patients with CGD, as observed in this study (9, 10, 12, 13, 41, 42). The diversity of microorganisms isolated in this study, despite the technical, operational, and logistical challenges of pathogen isolation, demonstrates that health systems should pay more attention to the socio-environmental context of patients with CGD in Latin America since the infectious profile has important implications for morbidity and mortality, as well as on consequences for future HSCT. The observed high prevalence of *Aspergillus* infections could be linked to environmental factors, while the occurrence of severe BCG vaccine reactions suggests a need to re-evaluate vaccination policies for CGD patients in endemic regions (8, 10, 12, 13, 16, 20).

The gastrointestinal involvement, particularly in cases of granulomatous colitis, highlights the need for multidisciplinary approaches to manage both infectious and inflammatory complications. IBD in CGD can affect up to 50% of patients and is a significant cause of weight and height deficit in these patients (21, 43). Overall, 31 patients presented IBD (13%) with or without granulomas, being more common among Brazilian patients with XL-CGD, who were mostly treated with corticosteroids, as they help reduce inflammation and also assist in the treatment of hepatic abscesses and granulomas, which occur in approximately one-third of CGD patients (2, 44). In cases of poor response to corticosteroids or hyperinflammation, immunobiologicals are especially effective (2, 43). Infliximab, a chimeric monoclonal antibody against tumor necrosis factor (TNF)-α, has shown promising results in persistent colitis associated with CGD and fistula closure; however, it can lead to an increase in the frequency of serious infections with typical CGD pathogens that can quickly progress to death (45, 46). Other anti-TNF antibodies, such as adalimumab and golimumab and the antagonist of the p40 subunit of interleukin (IL)-12 and IL-23 (ustekinumab), have been used in inflammatory bowel processes with good results (7, 47). Finally, HSCT leads to complete and stable colitis remission in patients with CGD (21, 23).

All patients received standard prophylaxis (cotrimoxazole and itraconazole). At the same time, IFN-γ was almost exclusively administered to patients from Mexico (89% of those with access). Therefore, it was not possible to compare it with other countries that made this drug available (Brazil and Argentina). IFN-γ has been shown to reduce the number and severity of infections and the relative risk of serious infections without significant adverse effects in prolonged use with a decrease in hospitalization time (21, 22). IFN-γ is currently accepted and administered in several countries to treat CGD (8, 9, 10, 13). Of note, in Brazil, this drug remains unregistered with the National Health Surveillance Agency, which limits access and dissemination of its use (only nine patients had access) (8).

HSCT outcomes varied, with a higher success rate in Brazilian patients, likely due to better access to specialized centers. Despite the curative potential of HSCT, the risk of complications remains high, highlighting the importance of careful patient selection and optimized transplantation protocols since this therapeutic option is frequently utilized when standard prophylaxis fails to control infections, resulting in patients being transplanted with significant sequelae from previous infections (2, 40, 45, 48). Expanding access to HSCT, the early transplantation and enhancing supportive care could improve outcomes for more patients throughout Latin America.

This study provides a comprehensive analysis of CGD in Latin America, detailing clinical and genetic characteristics and highlighting the disparities in healthcare access and outcomes across the region. Our findings emphasize the relevance of early diagnosis, access to genetic testing, and improved therapeutic options to enhance patient quality of life and survival rates. These needs become evident when we compare the survival curve of patients in Latin America with the curve of the study in the USA, which evaluated the survival of 227 patients with CGD based on residual O_2_^−^ production, showing a delay of more than 10 years for the first deaths, while in Latin America survival reached almost 70% in the same interval, reaching almost 50% at 25 years (49). The high frequency of novel pathogenic variants observed underscores the need for a Latin America-specific genetic database to aid in early diagnosis and personalized treatment planning.

Future efforts to develop region-specific guidelines, expand HSCT access, and implement newborn screening programs for CGD are essential steps for reduce the disease burden. This study provides the basis for future research and policy development to support CGD patients in Latin America and improve healthcare equity within this population. Continued collaborative efforts across the region will be essential to advance CGD treatment and ensure timely and effective interventions for affected patients.

## Materials and methods

### Data collection

Patient data were collected from the diagnostic and research service registry of the Human Immunology Laboratory at the Institute of Biomedical Sciences (ICB) at the USP and subsequently from the Latin American Society for Immunodeficiencies (LASID) registry after obtaining consent from the participating physicians and informed consent signed by the patients or their parents. Additional information was obtained through a detailed online questionnaire completed by the physicians, which included demographic, clinical, microbiological, laboratory, genetic, and family data (history of CGD, early death due to infection, and consanguinity), as well as information on treatment (prophylaxis and therapy, including HSTC), hospitalization, reactions to the BCG vaccine, TB, chronic inflammatory manifestations, and death. Data collection occurred between 2020 and 2022, using records from patients diagnosed between 1981 and 2021 from 53 pediatric hospitals in Brazil, Mexico, Chile, Costa Rica, Argentina, Paraguay, Peru, and Uruguay (30 of which were registered in LASID). After correcting divergent or duplicate data, the study included 238 patients. The LASID registry and the use of recorded information were approved by the National Health Council of the Brazilian Ministry of Health for international studies (CONEP 25000.040727/2008-16, CAAE 0034.1.146.000-08). Ethical approvals were obtained from all research ethics committees of the participating countries’ institutions, in accordance with the Helsinki Convention, as well as from the Research Ethics Committee of ICB-USP (CAAE: 39743920.5.0000.5467) and the Ethics Committee of the Hospital das Clínicas da Faculdade de Medicina da USP (CAAE: 39743920.5.3001.0068).

### Diagnostic criteria

The present study included patients of both sexes diagnosed with CGD, based on the identification of laboratory and clinical findings: (1) reduced or absent production of reactive oxygen species (ROS) or defective expression of components of the phagocyte NADPH oxidase complex (gp91^phox^, p22^phox^, p47^phox^, p67^phox^, p40^phox^, and EROS) (33, 50, 51, 52, 53); (2) identification of a pathogenic variant in any of the involved genes (*CYBB*, *CYBA*, *NCF1*, *NCF2*, *NCF4*, and *CYBC1*), either in homozygosity, compound heterozygosity (AR form), or hemizygosity (XL form—or heterozygosity in the case of symptomatic carrier females) (3, 4, 5, 7); and (3) individual or family history of CGD with frequent infections or early death from severe infection or granulomatous disease (brother, maternal uncle, or maternal cousin with XL-CGD) or mother with dual populations regarding ROS production—suggestive of being a carrier (52, 54).

### Neutrophil function: NBT and DHR tests

Laboratory diagnosis of CGD was performed for all patients (except for one with a prior genetic diagnosis). It was based on the abnormal oxidative burst in granulocytes, using either the NBT assay (50) or DHR assay, or both (51). The NBT test was conducted in some patients, mainly those diagnosed before the 1990s, with subsequent confirmation of diagnosis in many of them using the DHR assay. In total, 229 patients were diagnosed using DHR, eight using NBT, and one patient had only a genetic diagnosis. The NBT test assesses the reduction of NBT to formazan (a visible blue pigment) in neutrophils after stimulation. Cells from CGD patients do not reduce NBT, except in cases with residual ROS production (50). DHR, which has replaced NBT and is the current gold standard for CGD diagnosis, detects by flow cytometry the oxidation of DHR to rhodamine-123 (fluorescent) by ROS in stimulated neutrophils. The median fluorescence intensity of activated cells directly correlates with superoxide production (typically <5% of normal control), allowing not only for the diagnosis of the deficiency but also the inference of inheritance, carrier status in females, and the association of ROS levels with disease severity (33, 51, 52, 53).

### Genetic analysis

Each healthcare center provided information on the results of genetic and molecular diagnoses. Only Mexico provided information on the expression of NADPH oxidase subunits from a previous study that also included additional genetic analysis of the Mexican patients described here (13). For genetic investigation of Brazilian patients at ICB-USP, genomic deoxyribonucleic acid (gDNA) was extracted from peripheral blood using the Wizard Genomic DNA Purification Extraction Kit (Promega Corporation), after erythrocyte lysis according to the manufacturer’s instructions. The concentration and quality of the purified gDNA were assessed using a NanoDrop 2000 (Thermo Fisher Scientific), and DNA integrity was confirmed by electrophoresis on a 1.5% agarose gel (Sigma-Aldrich) stained with SYBR Green-II (Thermo Fisher Scientific). The exons and flanking intron sequences of the genes *CYBB*, *CYBA*, *NCF2*, and *NCF4* were amplified by polymerase chain reaction (primers and conditions used are available upon request) using Taq DNA polymerase (Life Technologies), and the amplicons were sent to the Human Genome Study Center (ICB-USP, São Paulo) for Sanger sequencing and whole-exome sequencing.

### Data analysis

The onset of symptoms was estimated based on data regarding the first relevant symptoms presented before diagnosing the oxidative burst deficiency by DHR or NBT. Diagnostic delay was calculated by subtracting the age at diagnosis from the age at onset of symptoms. Infectious and inflammatory clinical manifestations occurred over their lifetime, with etiological agents not always isolated but clinically inferred, with emphasis on pyogenic and mycobacterial infections, including reactions to the BCG vaccine, which were categorized into BCG-osis, BCG-itis, and local reaction. Confirmed cases of infection were quantified and evaluated regarding the main infectious sites. Information on treatment was limited to standard prophylaxis against infections, IFN-γ, corticosteroids, and HSCT. Genetic variants not found in specialized databases (e.g., HGMD, GnomAD, 1000 Genomes Project, and ClinVar) or in scientific articles were considered “novel” variants and assessed using pathogenicity predictors (CADD, SIFT, MutationTaster, and POLYPHEN-2). Hospitalization per year was calculated by dividing the total number of hospitalizations, regardless of cause, by the follow-up time in years and then compared between groups of patients with XL-CGD, AR-CGD + UGfem (unknown genetic females), and UG-CGD (unknown genetic CGD) using the Kruskal–Wallis test (P < 0.05). A similar analysis was performed, considering the cause of hospitalization, between the XL-CGD and AR-CGD + UGfem groups, using the Mann–Whitney test (P < 0.05). For survival analysis, it was necessary to group AR-CGD patients with female patients with UG, clinically diagnosed as AR-CGD, to obtain a statistically valid “*n*” (two patients [P145 and P205] carrying of a pathogenic variant in *CYBB*, were excluded from this group). The analysis period started from the date of birth and continued until the last follow-up update or the analyzed outcome (death). Two patients were not included in the survival analysis: one due to missing date of birth and another due to missing exact date of death. Mortality was assessed by crude rate (number of deaths in the group/number of individuals in the group × 100), specific rate (number of deaths from a specific cause/number of patients in the group × 100), and case fatality rate (deaths from specific infection/total number of patients with the same infection by group × 100). Comparison between groups was assessed using the χ^2^ test or Fisher’s exact test (95% CI) and the prevalence ratio and odds ratio (95% CI).

### Geospatial analysis

For the geospatial analysis, maps were created using Geographic Information System (GIS) technology and spatial statistics. In spatial statistics, the distribution of patient origins and diagnostic and follow-up centers in Brazil was explored and described using Quantum Geographic Information System (QGIS) software. The maps were referenced using the cartographic base obtained from the Instituto Brasileiro de Geografia e Estatística and equivalent institutions in other countries: Instituto Geográfico Nacional of Argentina, Instituto Nacional de Estadística (INE) of Paraguay, Instituto Nacional de Estadística e Informática of Peru, INE of Uruguay, INE of Chile, and Comisión Nacional para el Conocimiento y Uso de la Biodiversidad of Mexico. The geographic distribution of the IEIs network centers in Brazil was obtained from the LASID Registry and the detailed questionnaire. In contrast, for the other countries, the analysis was performed by geographic region of the patient’s origin due to the lack of precise identification of diagnostic and follow-up locations (geographic coordinates). Shapefiles (.shp), an Environmental System Research Institute vector data format for storing geographic coordinates, shape, and attributes of geographic features in GIS, were obtained from each of the described institutions, and, in QGIS software, the geographic location spreadsheet of each center (Brazil only) and patients was incorporated into the shapefile of each country (13, 55, 56). To represent the data, geometric shapes (triangles for medical centers and circles for patients) were used, with size determined by class.

### Online supplemental material

Table S1 shows the genetic characterization and clinical outcomes of all patients with CGD in this study (*n* = 238).

## Ethics statement

### Ethics approval

This study was performed in line with the principles of the Declaration of Helsinki. Approval was granted by the Ethics Committee of the ICB, USP, and by the Ethics Committee of Hospital das Clínicas da Faculdade de Medicina da Universidade de São Paulo - Children’s Institute (CAAE: 39743920.5.0000.5467 and 39743920.5.3001.0068).

### Consent to participate

Informed consent was obtained from all participants included in the study according to local ethics guidelines.

### Consent for publication

All authors agree to the publication of this manuscript.

## Supplementary Material

Table S1 shows the genetic characterization and clinical outcomes of all patients with CGD in this study (*n* = 238).

## Data Availability

The data are available from the corresponding author upon reasonable request.

## References

[1] Oliveira-Junior, E.B., J. Bustamante, P.E. Newburger, and A. Condino-Neto. 2011. The human NADPH oxidase: Primary and secondary defects impairing the respiratory burst function and the microbicidal ability of phagocytes. Scand. J. Immunol. 73:420–427. 10.1111/j.1365-3083.2010.02501.x 21204900

[2] Yu, H.-H., Y.-H. Yang, and B.-L. Chiang. 202. Chronic granulomatous disease: A comprehensive review. Clin. Rev. Allergy Immunol. 61:101–113. 10.1007/s12016-020-08800-x 32524254

[3] Roos, D., D.B. Kuhns, A. Maddalena, J. Roesler, J.A. Lopez, T. Ariga, T. Avcin, M. de Boer, J. Bustamante, A. Condino-Neto, . 2010. Hematologically important mutations: X-Linked chronic granulomatous disease (third update). Blood Cells Mol. Dis. 45:246–265. 10.1016/j.bcmd.2010.07.012 20729109 PMC4360070

[4] Roos, D., D.B. Kuhns, A. Maddalena, J. Bustamante, C. Kannengiesser, M. de Boer, K. van Leeuwen, M.Y. Köker, B. Wolach, J. Roesler, . 2010. Hematologically important mutations: The autosomal recessive forms of chronic granulomatous disease (second update). Blood Cells. Mol. Dis. 44:291–299. 10.1016/j.bcmd.2010.01.009 20167518 PMC4568122

[5] Arnadottir, G.A., G.L. Norddahl, S. Gudmundsdottir, A.B. Agustsdottir, S. Sigurdsson, B.O. Jensson, K. Bjarnadottir, F. Theodors, S. Benonisdottir, E.V. Ivarsdottir, . 2018. A homozygous loss-of-function mutation leading to CYBC1 deficiency causes chronic granulomatous disease. Nat. Commun. 9:4447. 10.1038/s41467-018-06964-x 30361506 PMC6202333

[6] Thomas, D.C., L.-M. Charbonnier, A. Schejtman, H. Aldhekri, E.L. Coomber, E.R. Dufficy, A.E. Beenken, J.C. Lee, S. Clare, A.O. Speak, . 2019. EROS/CYBC1 mutations: Decreased NADPH oxidase function and chronic granulomatous disease. J. Allergy Clin. Immunol. 143:782–785.e1. 10.1016/j.jaci.2018.09.019 30312704 PMC6490677

[7] van de Geer, A., A. Nieto-Patlán, D.B. Kuhns, A.T. Tool, A.A. Arias, M. Bouaziz, M. de Boer, J.L. Franco, R.P. Gazendam, J.L. van Hamme, . 2018. Inherited p40phox deficiency differs from classic chronic granulomatous disease. J. Clin. Invest. 128:3957–3975. 10.1172/JCI97116 29969437 PMC6118590

[8] de Oliveira-Junior, E.B., N.B. Zurro, C. Prando, O. Cabral-Marques, P.V.S. Pereira, L.-F. Schimke, S. Klaver, M. Buzolin, L. Blancas-Galicia, L. Santos-Argumedo, . 2015. Clinical and genotypic spectrum of chronic granulomatous disease in 71 Latin American patients: First report from the LASID registry. Pediatr. Blood Cancer. 62:2101–2107. 10.1002/pbc.25674 26185101

[9] Winkelstein, J.A., M.C. Marino, R.B. Johnston Jr, J. Boyle, J. Curnutte, J.I. Gallin, H.L. Malech, S.M. Holland, H. Ochs, P. Quie, . 2000. Chronic granulomatous disease. Report on a national registry of 368 patients. Medicine (Madr). 79:155–169. 10.1097/00005792-200005000-00003 10844935

[10] van den Berg, J.M., E. van Koppen, A. Ahlin, B.H. Belohradsky, E. Bernatowska, L. Corbeel, T. Español, A. Fischer, M. Kurenko-Deptuch, R. Mouy, . 2009. Chronic granulomatous disease: The European experience. PLoS One. 4:e5234. 10.1371/journal.pone.0005234 19381301 PMC2668749

[11] Wolach, B., R. Gavrieli, M. de Boer, K. van Leeuwen, S. Berger-Achituv, T. Stauber, J. Ben Ari, M. Rottem, Y. Schlesinger, G. Grisaru-Soen, . 2017. Chronic granulomatous disease: Clinical, functional, molecular, and genetic studies. The Israeli experience with 84 patients. Am. J. Hematol. 92:28–36. 10.1002/ajh.24573 27701760

[12] Rawat, A., P. Vignesh, M. Sudhakar, M. Sharma, D. Suri, A. Jindal, A. Gupta, J.K. Shandilya, S.K. Loganathan, G. Kaur, . 2021. Clinical, immunological, and molecular profile of chronic granulomatous disease: A multi-centric study of 236 patients from India. Front. Immunol. 12:625320. 10.3389/fimmu.2021.625320 33717137 PMC7946827

[13] Blancas-Galicia, L., E. Santos-Chávez, C. Deswarte, Q. Mignac, I. Medina-Vera, X. León-Lara, M. Roynard, S.C. Scheffler-Mendoza, R. Rioja-Valencia, A. Alvirde-Ayala, . 2020. Genetic, immunological, and clinical features of the first Mexican cohort of patients with chronic granulomatous disease. J. Clin. Immunol. 40:475–493. 10.1007/s10875-020-00750-5 32040803

[14] Chiu, T.L.-H., D. Leung, K.-W. Chan, H.M. Yeung, C.-Y. Wong, H. Mao, J. He, P. Vignesh, W. Liang, W.K. Liew, . 2021. Phenomic analysis of chronic granulomatous disease reveals more severe integumentary infections in X-linked compared with autosomal recessive chronic granulomatous disease. Front. Immunol. 12:803763. 10.3389/fimmu.2021.803763 35140711 PMC8818666

[15] Fattahi, F., M. Badalzadeh, L. Sedighipour, M. Movahedi, M.R. Fazlollahi, S.D. Mansouri, G.T. Khotaei, M.H. Bemanian, F. Behmanesh, A.A. Hamidieh, . 2011. Inheritance pattern and clinical aspects of 93 Iranian patients with chronic granulomatous disease. J. Clin. Immunol. 31:792–801. 10.1007/s10875-011-9567-x 21789723

[16] Conti, F., S.O. Lugo-Reyes, L. Blancas Galicia, J. He, G. Aksu, E. Borges de Oliveira Jr, C. Deswarte, M. Hubeau, N. Karaca, M. de Suremain, . 2016. Mycobacterial disease in patients with chronic granulomatous disease: A retrospective analysis of 71 cases. J. Allergy Clin. Immunol. 138:241–248.e3. 10.1016/j.jaci.2015.11.041 26936803

[17] Marciano, B.E., C.-Y. Huang, G. Joshi, N. Rezaei, B.C. Carvalho, Z. Allwood, A. Ikinciogullari, S.M. Reda, A. Gennery, V. Thon, . 2014. BCG vaccination in patients with severe combined immunodeficiency: Complications, risks, and vaccination policies. J. Allergy Clin. Immunol. 133:1134–1141. 10.1016/j.jaci.2014.02.028 24679470 PMC4015464

[18] Zhou, Q., X. Hui, W. Ying, J. Hou, W. Wang, D. Liu, Y. Wang, Y. Yu, J. Wang, J. Sun, . 2018. A cohort of 169 chronic granulomatous disease patients exposed to BCG vaccination: A retrospective study from a single center in Shanghai, China (2004-2017). J. Clin. Immunol. 38:260–272. 10.1007/s10875-018-0486-y 29560547

[19] World Health Organization . 2018. BCG vaccine: WHO position paper, february 2018 - recommendations. Vaccine. 36:3408–3410. 10.1016/j.vaccine.2018.03.009 29609965

[20] Fekrvand, S., R. Yazdani, P. Olbrich, A. Gennery, S.D. Rosenzweig, A. Condino-Neto, G. Azizi, H. Rafiemanesh, G. Hassanpour, N. Rezaei, . 2020 Apr. Primary immunodeficiency diseases and Bacillus calmette-guérin (BCG)-Vaccine-Derived complications: A systematic review. J. Allergy Clin. Immunol. Pract. 8:1371–1386. 10.1016/j.jaip.2020.01.038 32006723

[21] Marciano, B.E., R. Wesley, E.S. De Carlo, V.L. Anderson, L.A. Barnhart, D. Darnell, H.L. Malech, J.I. Gallin, and S.M. Holland. 2004. Long-term interferon-gamma therapy for patients with chronic granulomatous disease. Clin. Infect. Dis. 39:692–699. 10.1086/422993 15356785

[22] International Chronic Granulomatous Disease Cooperative Study Group . 1991. A controlled trial of interferon gamma to prevent infection in chronic granulomatous disease. N. Engl. J. Med. 324:509–516. 10.1056/NEJM199102213240801 1846940

[23] Kang, E.M., B.E. Marciano, S. DeRavin, K.A. Zarember, S.M. Holland, and H.L. Malech. 2011. Chronic granulomatous disease: Overview and hematopoietic stem cell transplantation. J. Allergy Clin. Immunol. 127:1319–1328. 10.1016/j.jaci.2011.03.028 21497887 PMC3133927

[24] Chiesa, R., J. Wang, H.-J. Blok, S. Hazelaar, B. Neven, D. Moshous, A. Schulz, M. Hoenig, F. Hauck, A. Al Seraihy, . 2020. Hematopoietic cell transplantation in chronic granulomatous disease: A study of 712 children and adults. Blood. 136:1201–1211. 10.1182/blood.2020005590 32614953

[25] Güngör, T., P. Teira, M. Slatter, G. Stussi, P. Stepensky, D. Moshous, C. Vermont, I. Ahmad, P.J. Shaw, J.M. Telles da Cunha, . 2014. Reduced-intensity conditioning and HLA-matched haematopoietic stem-cell transplantation in patients with chronic granulomatous disease: A prospective multicenter study. Lancet. 383:436–448. 10.1016/S0140-6736(13)62069-3 24161820

[26] Holland, S.M. 2013. Chronic granulomatous disease. Hematol. Oncol. Clin. North Am. 27:89–99. 10.1016/j.hoc.2012.11.002 23351990 PMC3558921

[27] Barese, C., S. Copelli, R. Zandomeni, M. Oleastro, M. Zelazko, and E.M. Rivas. 2004. X-Linked chronic granulomatous disease: First report of mutations in patients of Argentina. J. Pediatr. Hematol. Oncol. 26:656–660. 10.1097/01.mph.0000139455.29962.be 15454837

[28] Oliveira, A.F.B., A.C. Pastorino, M.d.B. Dorna, A.P.B.M. Castro, J.R.M. Pegler, B. Morgenstern, and M.M.S. Carneiro-Sampaio. 2021. Microbiological profile in chronic granulomatous disease patients in a single Brazilian primary immunodeficiency center. Allergol. Immunopathol. 49:217–224. 10.15586/aei.v49i2.82 33641311

[29] Heyworth, P.G., J.T. Curnutte, J. Rae, D. Noack, D. Roos, E. van Koppen, and A.R. Cross. 2001. Hematologically important mutations: X-Linked chronic granulomatous disease (second update). Blood Cells. Mol. Dis. 27:16–26. 10.1006/bcmd.2000.0347 11162142

[30] Rae, J., P.E. Newburger, M.C. Dinauer, D. Noack, P.J. Hopkins, R. Kuruto, and J.T. Curnutte. 1998. X-linked chronic granulomatous disease: Mutations in the CYBB gene encoding the gp91-phox component of respiratory-burst oxidase. Am. J. Hum. Genet. 62:1320–1331. 10.1086/301874 9585602 PMC1377153

[31] Wu, J., W.-F. Wang, Y.-D. Zhang, and T.-X. Chen. 2017. Clinical features and genetic analysis of 48 patients with chronic granulomatous disease in a single center study from Shanghai, China (2005-2015): New studies and a literature review. J. Immunol. Res. 2017:8745254. 10.1155/2017/8745254 28251166 PMC5303869

[32] Leusen, J.H., M. de Boer, B.G. Bolscher, P.M. Hilarius, R.S. Weening, H.D. Ochs, D. Roos, and A.J. Verhoeven. 1994. A point mutation in gp91-phox of cytochrome b558 of the human NADPH oxidase leading to defective translocation of the cytosolic proteins p47-phox and p67-phox. J. Clin. Invest. 93:2120–2126. 10.1172/JCI117207 8182143 PMC294341

[33] Berrón-Ruiz, L., A. Morín-Contreras, V. Cano-García, M.A. Yamazaki-Nakashimada, H. Gómez-Tello, M.E. Vargas-Camaño, R. Canseco-Raymundo, F. Saracho-Weber, D. Pietropaolo-Cienfuegos, B. Del Río-Navarro, . 2014. Detection of inheritance pattern in thirty-three Mexican males with chronic granulomatous disease through 123 dihydrorhodamine assay. Allergol. Immunopathol (Madr). 42:580–585. 10.1016/j.aller.2013.07.014 24890515

[34] López-Hernández, I., C. Deswarte, M.Á. Alcantara-Ortigoza, M.D.M. Saez-de-Ocariz, M.A. Yamazaki-Nakashimada, S.E. Espinosa-Padilla, J. Bustamante, and L. Blancas-Galicia. 2019. Skewed X-inactivation in a female carrier with X-linked chronic granulomatous disease. Iran. J. Allergy Asthma. Immunol. 18:447–451. 10.18502/ijaai.v18i4.1425 31522453

[35] Richards, S., N. Aziz, S. Bale, D. Bick, S. Das, J. Gastier-Foster, W.W. Grody, M. Hegde, E. Lyon, E. Spector, . 2015. Standards and guidelines for the interpretation of sequence variants: A joint consensus recommendation of the American College of medical genetics and genomics and the association for molecular pathology. Genet. Med. 17:405–424. 10.1038/gim.2015.30 25741868 PMC4544753

[36] Zwerling, A., M.A. Behr, A. Verma, T.F. Brewer, D. Menzies, and M. Pai. 2011. The BCG world atlas: A database of global BCG vaccination policies and practices. PLoS Med. 8:e1001012. 10.1371/journal.pmed.1001012 21445325 PMC3062527

[37] Gallin, J.I., D.W. Alling, H.L. Malech, R. Wesley, D. Koziol, B. Marciano, E.M. Eisenstein, M.L. Turner, E.S. DeCarlo, J.M. Starling, and S.M. Holland. 2003. Itraconazole to prevent fungal infections in chronic granulomatous disease. N. Engl. J. Med. 348:2416–2422. 10.1056/NEJMoa021931 12802027

[38] Tajik, S., M. Badalzadeh, M.R. Fazlollahi, M. Houshmand, N. Bazargan, M. Movahedi, M. Mahlouji Rad, S.A. Mahdaviani, S. Mamishi, G.T. Khotaei, . 2019. Genetic and molecular findings of 38 Iranian patients with chronic granulomatous disease caused by p47-phox defect. Scand. J. Immunol. 90:e12767. 10.1111/sji.12767 30963593

[39] Marciano, B.E., C. Spalding, A. Fitzgerald, D. Mann, T. Brown, S. Osgood, L. Yockey, D.N. Darnell, L. Barnhart, J. Daub, . 2015. Common severe infections in chronic granulomatous disease. Clin. Infect. Dis. 60:1176–1183. 10.1093/cid/ciu1154 25537876 PMC4400412

[40] Cole, T., M.S. Pearce, A.J. Cant, C.M. Cale, D. Goldblatt, and A.R. Gennery. 2013. Clinical outcome in children with chronic granulomatous disease managed conservatively or with hematopoietic stem cell transplantation. J. Allergy Clin. Immunol. 132:1150–1155. 10.1016/j.jaci.2013.05.031 23870668

[41] Martire, B., R. Rondelli, A. Soresina, C. Pignata, T. Broccoletti, A. Finocchi, P. Rossi, M. Gattorno, M. Rabusin, C. Azzari, . 2008. Clinical features, long-term follow-up and outcome of a large cohort of patients with chronic granulomatous disease: An Italian multicenter study. Clin. Immunol. 126:155–164. 10.1016/j.clim.2007.09.008 18037347

[42] Kuhns, D.B., A.P. Hsu, D. Sun, K. Lau, D. Fink, P. Griffith, D.W. Huang, D.A.L. Priel, L. Mendez, S. Kreuzburg, . 2019. NCF1 (p47phox)-deficient chronic granulomatous disease: Comprehensive genetic and flow cytometric analysis. Blood Adv. 3:136–147. 10.1182/bloodadvances.2018023184 30651282 PMC6341190

[43] Magnani, A., P. Brosselin, J. Beauté, N. de Vergnes, R. Mouy, M. Debré, F. Suarez, O. Hermine, O. Lortholary, S. Blanche, . 2014. Inflammatory manifestations in a single-center cohort of patients with chronic granulomatous disease. J. Allergy Clin. Immunol. 134:655–662.e8. 10.1016/j.jaci.2014.04.014 24985400

[44] Straughan, D.M., K.C. McLoughlin, J.E. Mullinax, B.E. Marciano, A.F. Freeman, V.L. Anderson, G. Uzel, S.C. Azoury, R. Sorber, H.S. Quadri, . 2018. The changing paradigm of management of liver abscesses in chronic granulomatous disease. Clin. Infect. Dis. 66:1427–1434. 10.1093/cid/cix1012 29145578 PMC6248449

[45] Leiding, J.W., and S.M. Holland. 2012. Chronic Granulomatous Disease. *In* GeneReviews. M.P. Adam, D.B. Everman, G.M. Mirzaa, R.A. Pagon, S.E. Wallace, L.J.H. Bean, K.W. Gripp, and A. Amemiya, editors. University of Washington, Seattle, Seattle (WA). 1993–2022. 22876374

[46] Uzel, G., J.S. Orange, N. Poliak, B.E. Marciano, T. Heller, and S.M. Holland. 2010. Complications of tumor necrosis factor-α blockade in chronic granulomatous disease-related colitis. Clin. Infect. Dis. 51:1429–1434. 10.1086/657308 21058909 PMC3106244

[47] Sands, B.E., W.J. Sandborn, R. Panaccione, C.D. O’Brien, H. Zhang, J. Johanns, O.J. Adedokun, K. Li, L. Peyrin-Biroulet, G. Van Assche, . 2019. Ustekinumab as induction and maintenance therapy for ulcerative colitis. N. Engl. J. Med. 381:1201–1214. 10.1056/NEJMoa1900750 31553833

[48] Horwitz, M.E., A.J. Barrett, M.R. Brown, C.S. Carter, R. Childs, J.I. Gallin, S.M. Holland, G.F. Linton, J.A. Miller, S.F. Leitman, . 2001. Treatment of chronic granulomatous disease with nonmyeloablative conditioning and a T-cell-depleted hematopoietic allograft. N. Engl. J. Med. 344:881–888. 10.1056/NEJM200103223441203 11259721

[49] Kuhns, D.B., W.G. Alvord, T. Heller, J.J. Feld, K.M. Pike, B.E. Marciano, G. Uzel, S.S. DeRavin, D.A.L. Priel, B.P. Soule, . 2010. Residual NADPH oxidase and survival in chronic granulomatous disease. N. Engl. J. Med. 363:2600–2610. 10.1056/NEJMoa1007097 21190454 PMC3069846

[50] Roos, D., and M. de Boer. 2014. Molecular diagnosis of chronic granulomatous disease. Clin. Exp. Immunol. 175:139–149. 10.1111/cei.12202 24016250 PMC3892405

[51] Elloumi, H.Z., and S.M. Holland. 2007. Diagnostic assays for chronic granulomatous disease and other neutrophil disorders. Methods Mol. Biol. 412:505–523. 10.1007/978-1-59745-467-4_31 18453131

[52] Marciano, B.E., C.S. Zerbe, E.L. Falcone, L. Ding, S.S. DeRavin, J. Daub, S. Kreuzburg, L. Yockey, S. Hunsberger, L. Foruraghi, . 2018. X-linked carriers of chronic granulomatous disease: Illness, lyonization, and stability. J. Allergy Clin. Immunol. 141:365–371. 10.1016/j.jaci.2017.04.035 28528201

[53] Yu, J.E., A.E. Azar, H.J. Chong, A.M. Jongco 3rd, and B.T. Prince. 2018. Considerations in the diagnosis of chronic granulomatous disease. J. Pediatr. Infect. Dis. Soc. 7:S6–S11. 10.1093/jpids/piy007 PMC5946934 29746674

[54] Tangye, S.G., W. Al-Herz, A. Bousfiha, C. Cunningham-Rundles, J.L. Franco, S.M. Holland, C. Klein, T. Morio, E. Oksenhendler, C. Picard, . 2022. Human inborn errors of immunity: 2022 update on the classification from the international union of immunological societies expert committee. J. Clin. Immunol. 42:1473–1507. 10.1007/s10875-022-01289-3 35748970 PMC9244088

[55] Boton Pereira, D.H., L.S. Primo, G. Pelizari, E. Flores, D. de Moraes-Vasconcelos, A. Condino-Neto, and L.E. Prestes-Carneiro. 2020. Primary Immunodeficiencies in a mesoregion of São Paulo, Brazil: Epidemiologic, clinical, and geospatial approach. Front. Immunol. 11:862. 10.3389/fimmu.2020.00862 32477349 PMC7235164

[56] Oliveira, T.S.de, S.J.M.S. da, R.M.R. Silva, M.S. Moreau, A.P.M. Mariano, and M.F.da. Silva. 2020. Geospatial and transmission evidences of the human fascioliasis cases (2012-2017) in south bahia, Brazil/Evidências Geoespaciais e de Transmissão de Casos de Fasciolíase Humana (2012-2017) no Sul da Bahia, Brasil. Braz. J. Dev. 6:13786–13801. 10.34117/bjdv6n3-298

