# Supplementary material for: Clinical features, genotypes, and geographic distribution of 238 Latin American CGD patients
Source: J Hum Immun. 2025 Oct 6;1(4):e20250033. doi: 10.70962/jhi.20250033 (PMC13177377; doi:10.70962/jhi.20250033)
Supplement: Table S1 — shows the genetic characterization and clinical outcomes of all patients with CGD in this study (n = 238). [file jhi_20250033_tables1.docx]

| Supplementary table S1 Genetic characterization and clinical outcomes of all patients with CGD in this study (n = 238). | | | | | | | | | | | | |
| --- | --- | --- | --- | --- | --- | --- | --- | --- | --- | --- | --- | --- |
| ID | **Fam.** | **Count.** | **Sex** | **Gene** | **Site** | **Pathogenic variant** | **Protein** | **Type** | **Zygo.** | **Express.** | **Outcome** | **Ref.** |
| P1 | A | BRA | M | *CYBB* | ex. 9 | c.1158delG | p.Trp380* | *Nonsense* | Hemi | − | Alive | ND |
| P2 | B | BRA | M | UnK. | − | − | − | − | − | − | Alive | − |
| P3 | Csi | BRA | M | *CYBB* | ex. 2 | c.125C>A | p.Thr42Lis | *Missense* | Hemi | − | Dead | [3] |
| P4 | Csi | BRA | M | UnK. | − | − | − | − | − | − | Alive | − |
| P5 | Dsi | BRA | M | *NCF1* | ex. 2 | c.75_76delGT | p.Tyr26Hisfs*26 | *Deletion* | Homo | A47^0^ | Alive | [4] |
| P6 | Dsi | BRA | F | *NCF1* | ex. 2 | c.75_76delGT | p.Tyr26Hisfs*26 | *Deletion* | Homo | A47^0^ | Dead | [4] |
| P7 | E | BRA | M | UnK. | − | − | − | − | − | − | Alive | − |
| P8 | F | BRA | M | UnK. | − | − | − | − | − | − | Alive | − |
| P9 | G | BRA | M | UnK. | − | − | − | − | − | − | Dead | − |
| P10 | H | BRA | M | UnK. | − | − | − | − | − | − | Dead | − |
| P11 | I | BRA | M | UnK. | − | − | − | − | − | − | Dead | − |
| P12 | J | BRA | M | UnK. | − | − | − | − | − | − | Alive | − |
| P13 | K | BRA | F | UnK. | − | − | − | − | − | − | Alive | − |
| P14 | L | BRA | M | UnK. | − | − | − | − | − | − | Alive | − |
| P15 | M | BRA | M | UnK. | − | − | − | − | − | − | Alive | − |
| P16 | N | BRA | F | *NCF1* | ex. 2 | c.75_76delGT | p.Tyr26Hisfs*26 | *Deletion* | Homo | A47^0^ | Alive | [4] |
| P17 | O | BRA | M | *CYBB* | ex. 9 | c.1022C>T | p.Thr341Ile | *Missense* | Hemi | X91^−^ | Alive | [3, 50] |
| P18 | P | BRA | M | *CYBB* | ex. 6 | c.665A>T | p.His222Leu | *Missense* | Hemi | X91^?^ | Dead | [3] |
| P19 | Qc | BRA | M | *CYBB* | ex. 3 | c.217C>T | p.Arg73* | *Nonsense* | Hemi | X91^0^ | Dead | [3, 50] |
| P20 | Rsi | BRA | M | *CYBB* | ex. 1 | c.12_15delGGCT | p.Ala5* | *Nonsense* | Hemi | − | Dead | [51] |
| P21 | Rsi | BRA | M | *CYBB* | ex. 1 | c.12_15delGGCT | p.Ala5* | *Nonsense* | Hemi | − | Dead | [51] |
| P22 | S | BRA | M | UnK. | − | − | − | − | − | − | Alive | − |
| P23 | Qc | BRA | M | *CYBB* | ex. 3 | c.217C>T | p.Arg73* | *Nonsense* | Hemi | X91^0^ | Alive | [3, 50] |
| P24 | T | BRA | M | UnK. | − | − | − | − | − | − | Alive | − |
| P25 | Qc | BRA | M | *CYBB* | ex. 3 | c.217C>T | p.Arg73* | *Nonsense* | Hemi | X91^0^ | Alive | [3, 50] |
| P26 | U | BRA | M | UnK. | − | − | − | − | − | − | Alive | − |
| P27 | V | BRA | M | *CYBB* | ex. 5 | c.375G>A | p.Trp125* | *Nonsense* | Hemi | X91^?^ | Alive | [3] |
| P28 | W | BRA | M | UnK. | − | − | − | − | − | − | Alive | − |
| P29 | X | BRA | M | *CYBB* | ex. 7 | c.752G>A | p.Trp251* | *Nonsense* | Hemi | X91^0^ | Dead | [3, 50] |
| P30 | Y | BRA | M | UnK. | − | − | − | − | − | − | Alive | − |
| P31 | Z | BRA | M | UnK. | − | − | − | − | − | − | Alive | − |
| P32 | AA | BRA | F | UnK. | − | − | − | − | − | − | Alive | − |
| P33 | AB | BRA | F | *NCF1* | ex. 2 | c.75_76delGT | p.Tyr26Hisfs*26 | *Deletion* | Homo | A47^0^ | Alive | [4] |
| P34 | AC | BRA | F | UnK. | − | − | − | − | − | − | Alive | − |
| P35 | AD | BRA | M | *CYBB* | ex. 8 | c.868C>T | p.Arg290* | *Nonsense* | Hemi | X91^0^ | Alive | [3, 50] |
| P36 | AE | BRA | M | UnK. | − | − | − | − | − | − | Alive | − |
| P37 | AF | BRA | M | UnK. | − | − | − | − | − | − | Alive | − |
| P38 | AG | BRA | F | UnK. | − | − | − | − | − | − | Alive | − |
| P39 | AH | BRA | M | *CYBB* | ex. 5 | c.430C>T | p.Leu144Phe | *Missense* | Hemi | X91^?^ | Alive | ND |
| P40 | AI | BRA | M | *CYBB* | ex. 7 | c.676C>T | p.Arg226* | *Nonsense* | Hemi | X91^0^ | Alive | [3, 50] |
| P41 | AJ | BRA | M | *CYBB* | ex. 13 | c.1679delG | p.Gly560Glufs*17 | *Deletion* | Hemi | X91^0^ | Alive | [3] |
| P42 | AK | BRA | M | UnK. | − | − | − | − | − | − | Dead | − |
| P43 | AL | BRA | F | *NCF1* | ex. 2 | c.75_76delGT | p.Tyr26Hisfs*26 | *Deletion* | Homo | A47^0^ | Alive | [4] |
| P44 | AM | BRA | M | UnK. | − | − | − | − | − | − | Alive | − |
| P45 | AN | BRA | F | UnK. | − | − | − | − | − | − | Alive | − |
| P46 | AO | BRA | M | UnK. | − | − | − | − | − | − | Alive | − |
| P47 | AP | BRA | M | *CYBB* | ex. 5 | c.388C>T | p.Arg130* | *Nonsense* | Hemi | X91^0^ | Alive | [3, 50] |
| P48 | AQ | BRA | M | *CYBB* | ex. 9 | c.1140G>A | p.Trp380* | *Nonsense* | Hemi | X91^0^ | Alive | [3] |
| P49 | AR | BRA | F | UnK. | − | − | − | − | − | − | Alive | − |
| P50 | ASsi | BRA | F | *NCF1* | ex. 2 | c.75_76delGT | p.Tyr26Hisfs*26 | *Deletion* | Homo | A47^0^ | Alive | [4] |
| P51 | ASsi | BRA | F | *NCF1* | ex. 2 | c.75_76delGT | p.Tyr26Hisfs*26 | *Deletion* | Homo | A47^0^ | Alive | [4] |
| P52 | AT | BRA | M | *CYBB* | ex. 13 | c.1609T>C | p.Cys537Arg | *Missense* | Hemi | X91^+^ | Alive | [3, 50] |
| P53 | AUsi | BRA | F | UnK. | − | − | − | − | − | − | Alive | − |
| P54 | AUsi | BRA | F | UnK. | − | − | − | − | − | − | Dead | − |
| P55 | AV | BRA | F | UnK. | − | − | − | − | − | − | Dead | − |
| P56 | AW | BRA | M | UnK. | − | − | − | − | − | − | Alive | − |
| P57 | AX | BRA | F | UnK. | − | − | − | − | − | − | Alive | − |
| P58 | AYsi | BRA | M | UnK. | − | − | − | − | − | − | Alive | − |
| P59 | AYsi | BRA | M | UnK. | − | − | − | − | − | − | Dead | − |
| P60 | AZ | BRA | F | *CYBA* | ex. 6 / ex. 5 | c.472_484del / c.399delC | p.Pro160Alafs*27 / p.Ile134Serfs*57 | *Deletion* / *Deletion* | Comp. | A22^?^ / A22^?^ | Alive | [4] |
| P61 | BAc | BRA | M | UnK. | − | − | − | − | − | − | Alive | − |
| P62 | BAc | BRA | M | *CYBB* | ex. 7 | c.754G>T | p.Gly252* | *Nonsense* | Hemi | X91^?^ | Alive | [3] |
| P63 | BB | BRA | M | *CYBB* | del. ex. 1_13 | del. ex. 1_13 | − | *Large del. (≥1 ex.)* | Hemi | X91^0^ | Alive | ND |
| P64 | BC | BRA | M | UnK. | − | − | − | − | − | − | Alive | − |
| P65 | BD | BRA | M | UnK. | − | − | − | − | − | − | Dead | − |
| P66 | BE | BRA | F | *NCF1* | ex. 2 | c.75_76delGT | p.Tyr26Hisfs*26 | *Deletion* | Homo | A47^0^ | Alive | [4] |
| P67 | BF | BRA | M | UnK. | − | − | − | − | − | − | Alive | − |
| P68 | BG | BRA | F | UnK. | − | − | − | − | − | − | Alive | − |
| P69 | BH | BRA | F | *NCF1* | ex. 2 | c.75_76delGT | p.Tyr26Hisfs*26 | *Deletion* | Homo | A47^0^ | Alive | [4] |
| P70 | BI | BRA | M | *CYBB* | del. ex. 5 | c.483G>A | del. ex. 5 (p.Lys161=) | *Splicing* | Hemi | X91^0^ | Alive | [3] |
| P71 | BJ | BRA | M | *CYBB* | ex. 5 | c.376T>C | p.Cis126Arg | *Missense* | Hemi | X91^?^ | Alive | [49] |
| P72 | BK | BRA | M | UnK. | − | − | − | − | − | − | Alive | − |
| P73 | BL | BRA | F | *NCF1* | ex. 2 | c.75_76delGT | p.Tyr26Hisfs*26 | *Deletion* | Homo | A47^0^ | Alive | [4] |
| P74 | BM | BRA | F | UnK. | − | − | − | − | − | − | Alive | − |
| P75 | BN | BRA | M | *CYBB* | ex. 9 | c.1010G>A | p.Trp337* | *Nonsense* | Hemi | X91^0^ | Alive | [3, 50] |
| P76 | BO | BRA | F | UnK. | − | − | − | − | − | − | Alive | − |
| P77 | BP | BRA | M | *CYBB* | del. ex. 3 | c.252G>A | del. ex. 3 (p.Ser48_Ala84del) | *Splicing* | Hemi | X91^0^ | Alive | [3, 50] |
| P78 | BQ | BRA | M | UnK. | − | − | − | − | − | − | Alive | − |
| P79 | BAc | BRA | M | UnK. | − | − | − | − | − | − | Dead | − |
| P80 | BR | BRA | M | UnK. | − | − | − | − | − | − | Alive | − |
| P81 | BAc | BRA | M | UnK. | − | − | − | − | − | − | Alive | − |
| P82 | BSsi | BRA | M | UnK. | − | − | − | − | − | − | Alive | − |
| P83 | BT | BRA | M | UnK. | − | − | − | − | − | − | Alive | − |
| P84 | BU | BRA | M | UnK. | − | − | − | − | − | − | Dead | − |
| P85 | BV | BRA | M | UnK. | − | − | − | − | − | − | Alive | − |
| P86 | BWsi | BRA | M | UnK. | − | − | − | − | − | − | Dead | − |
| P87 | BX | BRA | M | UnK. | − | − | − | − | − | − | Alive | − |
| P88 | BY | BRA | M | UnK. | − | − | − | − | − | − | Alive | − |
| P89 | BZ | BRA | M | UnK. | − | − | − | − | − | − | Dead | − |
| P90 | BSsi | BRA | M | UnK. | − | − | − | − | − | − | Alive | − |
| P91 | CA | BRA | M | UnK. | − | − | − | − | − | − | Alive | − |
| P92 | BWsi | BRA | M | UnK. | − | − | − | − | − | − | Alive | − |
| P93 | CB | BRA | M | UnK. | − | − | − | − | − | − | Alive | − |
| P94 | CC | BRA | M | UnK. | − | − | − | − | − | − | Dead | − |
| P95 | CD | BRA | M | UnK. | − | − | − | − | − | − | Alive | − |
| P96 | CE | BRA | F | UnK. | − | − | − | − | − | − | Alive | − |
| P97 | CF | MEX | M | *CYBB* | ex. 3 | c.217C>T | p.Arg73* | *Nonsense* | Hemi | X91^0^ | Dead | [3, 50] |
| P98 | CG | MEX | M | *CYBB* | ex. 13 | − | p.Ile532* | *Nonsense* | Hemi | X91^0^ | Alive | [13] |
| P99 | CH | MEX | F | UnK. | − | − | − | − | − | − | Dead | − |
| P100 | CI | MEX | M | UnK. | − | − | − | − | − | − | Dead | − |
| P101 | CJ | MEX | M | *NCF1* | ex. 2 | c.75_76delGT | p.Tyr26Hisfs*26 | *Deletion* | Homo | A47^0^ | Alive | [4] |
| P102 | CK | MEX | M | *CYBB* | ex. 6 | c.626A>G | p.His209Arg | *Missense* | Hemi | X91^0^ | Alive | [13] |
| P103 | CL | MEX | M | *CYBB* | ex. 9 | c.987C>A | p.Cys329* | *Nonsense* | Hemi | X91^?^ | Dead | [3] |
| P104 | CM | MEX | M | *CYBB* | ex. 12 | c.1499A>T | p.Asp500Val | *Missense* | Hemi | X91^+^ | Alive | [52] |
| P105 | CN | MEX | M | *CYBB* | ex. 6 | c.602dup | p.Tyr201* | *Insertion* | Hemi | X91^0^ | Dead | [13, 33] |
| P106 | CO | MEX | M | UnK. | − | − | − | − | − | − | Dead | − |
| P107 | CPc | MEX | M | *CYBA* | ex. 1 | c.4_24del21 | p.Gly2_Met8del | *Deletion* | Homo | A22^0^ | Alive | [13] |
| P108 | CQ | MEX | M | UnK. | − | − | − | − | − | − | Dead | − |
| P109 | CR | MEX | M | *CYBB* | ex. 12 | c.1545del | p.Trp516Glyfs*17 | *Deletion* | Hemi | X91^?^ | Dead | [13] |
| P110 | CSn | MEX | M | *CYBB* | ex. 9 | c.1016C>A | p.Pro339His | *Missense* | Hemi | X91^0^ | Dead | [13, 3, 50] |
| P111 | CT | MEX | M | *CYBB* | ex. 8 | c. 850_851delAG | p.Arg84Valfs*63 | *Deletion* | Hemi | X91^0^ | Dead | [13, 33] |
| P112 | CU | MEX | M | *CYBB* | ex. 2 | c.83G>A | p.Trp28* | *Nonsense* | Hemi | X91^0^ | Alive | [3] |
| P113 | CVsi | MEX | M | *CYBB* | ex. 7 | c.676C>T | p.Arg226* | *Nonsense* | Hemi | X91^0^ | Dead | [13, 3, 50] |
| P114 | CVsi | MEX | M | *CYBB* | ex. 7 | c.676C>T | p.Arg226* | *Nonsense* | Hemi | X91^0^ | Alive | [13, 3, 50] |
| P115 | CWsi | MEX | F | *NCF1* | ex. 2 | c.75_76delGT | p.Tyr26Hisfs*26 | *Deletion* | Homo | A47^0^ | Alive | [4] |
| P116 | CWsi | MEX | F | *NCF1* | ex. 2 | c.75_76delGT | p.Tyr26Hisfs*26 | *Deletion* | Homo | A47^0^ | Alive | [4] |
| P117 | CX | MEX | F | *CYBA* | in. 5 | c.370-1G>A | Splicing site | *Splicing* | Homo | A22^0^ | Alive | [13] |
| P118 | CY | MEX | F | *NCF1* | ex. 2 | c.75_76delGT | p.Tyr26Hisfs*26 | *Deletion* | Homo | A47^0^ | Alive | [4] |
| P119 | CZ | MEX | M | *CYBB* | ex. 6 | c.616T>C | p.Trp206Arg | *Missense* | Hemi | X91^?^ | Alive | ND |
| P120 | DA | MEX | M | *CYBB* | ex. 4 | c.277C>T | p.Gln93* | *Nonsense* | Hemi | X91^?^ | Dead | [8] |
| P121 | DB | MEX | M | UnK. | − | − | − | − | − | − | Dead | − |
| P122 | DC | MEX | F | UnK. | − | − | − | − | − | − | Dead | − |
| P123 | DD | MEX | F | UnK. | − | − | − | − | − | − | Dead | − |
| P124 | DEsi | MEX | M | *CYBB* | ex. 5 | c.425_426delCT | p.Ser142* | *Nonsense* | Hemi | X91^0^ | Alive | [3] |
| P125 | DF | MEX | M | UnK. | − | − | − | − | − | − | Alive | − |
| P126 | DGu | MEX | M | *CYBB* | ex. 9 | c.978delT | p.Phe326Leufs*17 | *Deletion* | Hemi | X91^?^ | Dead | ND |
| P127 | DEsi | MEX | M | *CYBB* | ex. 5 | c.425_426delCT | p.Ser142* | *Nonsense* | Hemi | X91^0^ | Alive | [3] |
| P128 | DHsi | MEX | F | UnK. | − | − | − | − | − | − | Dead | − |
| P129 | DHsi | MEX | M | UnK. | − | − | − | − | − | − | Dead | − |
| P130 | DI | MEX | M | UnK. | − | − | − | − | − | − | Dead | − |
| P131 | DJ | MEX | M | UnK. | − | − | − | − | − | − | Dead | − |
| P132 | DK | MEX | M | UnK. | − | − | − | − | − | − | Dead | − |
| P133 | DL | MEX | M | UnK. | − | − | − | − | − | − | Alive | − |
| P134 | DM | MEX | M | UnK. | − | − | − | − | − | − | Dead | − |
| P135 | DN | MEX | M | *CYBB* | ex. 9 | c.1019T>C | p.Phe340Ser | *Missense* | Hemi | X91^?^ | Dead | ND |
| P136 | DOsi | MEX | M | *CYBB* | ex. 11 | c.1447T>C | p.Trp483Arg | *Missense* | Hemi | X91^?^ | Dead | [8] |
| P137 | DP | MEX | M | *CYBB* | ex. 9 | c.1011G>A | p.Trp337* | *Nonsense* | Hemi | X91^0^ | Dead | [4] |
| P138 | DQ | MEX | M | UnK. | − | − | − | − | − | − | Dead | − |
| P139 | DR | MEX | M | *CYBB* | ex. 12 | c.1571C>T | p.Ala524Val | *Missense* | Hemi | X91^0^ | Dead | [3] |
| P140 | DS | MEX | M | UnK. | − | − | − | − | − | − | Dead | − |
| P141 | DT | MEX | M | *CYBB* | ex. 9 | c.1006G>T | p.Glu336* | *Nonsense* | Hemi | X91^0^ | Dead | [3, 50] |
| P142 | DU | MEX | M | *CYBB* | ex. 1 | c.12G>A | p.Trp4* | *Nonsense* | Hemi | X91^0^ | Dead | [3, 50] |
| P143 | DV | MEX | M | UnK. | − | − | − | − | − | − | Dead | − |
| P144 | DW | MEX | M | *CYBB* | in. 6 | c.675-12T>G | Splicing site | *Splicing* | Hemi | X91^?^ | Dead | [13, 33] |
| P145 | DXm | MEX | F | *CYBB* | ex. 7 | c.676C>T | p.Arg226* | *Nonsense* | Hetero | X91^0^ # | Alive | [48] |
| P146 | DY | MEX | M | *CYBB* | ex. 1 | c.12G>A | p.Trp4* | *Nonsense* | Hemi | X91^0^ | Dead | [3, 50] |
| P147 | DZsi | MEX | M | *CYBB* | del. ex. 1_13 | del. ex. 1_13 | − | *Large del. (≥1 ex.)* | Hemi | X91^0^ | Dead | [13] |
| P148 | DOsi | MEX | M | *CYBB* | ex. 11 | c.1447T>C | p.Trp483Arg | *Missense* | Hemi | X91^?^ | Alive | [8] |
| P149 | EA | MEX | M | *CYBB* | ex. 7 | c.722_726delTAACA | p.Ile241Serfs*3 | *Deletion* | Hemi | X91^0^ | Alive | [13, 33] |
| P150 | EB | MEX | M | *CYBB* | del. ex. 1_13 | del. ex. 1_13 + McLeod | − | *Large del. (≥1 ex.)* | Hemi | X91^0^ + MacLeod | Alive | [13, 33] |
| P151 | DZsi | MEX | M | *CYBB* | del. ex. 1_13 | del. ex. 1_13 | − | *Large del. (≥1 ex.)* | Hemi | X91^0^ | Alive | [13] |
| P152 | EC | MEX | M | *CYBB* | ex. 5 | c.345C>G | p.His115Gln | *Missense* | Hemi | X91^−^ | Alive | [13, 50] |
| P153 | ED | MEX | M | *CYBB* | del. ex. 1_13 | del. ex. 1_13 | − | *Large del. (≥1 ex.)* | Hemi | X91^0^ | Dead | [13] |
| P154 | EEsi | MEX | M | *CYBB* | ex. 13 | c.1612G>T | p.Gly538* | *Nonsense* | Hemi | X91^0^ | Dead | [13] |
| P155 | EEsi | MEX | M | *CYBB* | ex. 13 | c.1612G>T | p.Gly538* | *Nonsense* | Hemi | X91^0^ | Alive | [13] |
| P156 | EF | MEX | M | *CYBB* | ex. 13 | c.1678G>T | p.Gly560* | *Nonsense* | Hemi | X91^?^ | Alive | [3] |
| P157 | EG | MEX | M | *NCF1* | ex. 2 | c.75_76delGT | p.Tyr26Hisfs*26 | *Deletion* | Homo | A47^0^ | Dead | [4] |
| P158 | EH | MEX | M | *CYBB* | ex. 5 | c.374G>A | p.Trp125* | *Nonsense* | Hemi | X91^?^ | Dead | [3] |
| P159 | EI | MEX | M | *NCF1* | ex. 2 | c.75_76delGT | p.Tyr26Hisfs*26 | *Deletion* | Homo | A47^0^ | Alive | [4] |
| P160 | EJ | MEX | M | *CYBB* | ex. 9 | c.1085C>G | p.Thr362Arg | *Missense* | Hemi | X91^?^ | Alive | [3] |
| P161 | EK | MEX | M | *CYBB* | ex. 3 | c.142-1G>A | p.Ser48_Ala84del (del. ex. 3?) | *Splicing* | Hemi | X91^?^ | Dead | [3] |
| P162 | EL | MEX | F | *NCF2* | ex. 2 / ex. 5 | c.55_63del / c.661C>T | p.Lys19_Asp21del / p.Gln221* | *Deletion* / *Nonsense* | Comp. | A67^0^ / A67^?^ | Alive | [13, 4] |
| P163 | EM | MEX | M | *NCF2* | ex. 2 | c.55_63del | p.Lys19_Asp21del | *Deletion* | Homo | A67^0^ | Dead | [4] |
| P164 | EN | MEX | M | *CYBB* | ex. 9 | c.1148C>T | p.Pro383Leu | *Missense* | Hemi | X91^?^ | Alive | [16] |
| P165 | EO | MEX | M | *CYBB* | ex. 5 | c.388C>T | p.Arg130* | *Nonsense* | Hemi | X91^0^ | Alive | [3, 49, 50] |
| P166 | EP | MEX | M | *CYBB* | ex. 6 | c.626A>G | p.His209Arg | *Missense* | Hemi | X91^0^ | Dead | [13, 3, 50] |
| P167 | EQ | MEX | M | *NCF2* | ex. 2 | c.175delG | p.Ala59Profs*40 | *Deletion* | Homo | A67^?^ | Alive | ND |
| P168 | ER | MEX | M | UnK. | − | − | − | − | − | − | Dead | − |
| P169 | ES | MEX | M | *CYBB* | ex. 12 | c.1473del | p.Phe491Leufs*11 | *Deletion* | Hemi | X91^?^ | Dead | [13] |
| P170 | DXso | MEX | M | *CYBB* | ex. 7 | c.676C>T | p.Arg226* | *Nonsense* | Hemi | X91^0^ | Dead | [13, 3, 50] |
| P171 | ET | MEX | M | *CYBB* | ex. 6 | − | p.Trp206* | *Nonsense* | Hemi | X91^0^ | Dead | [13] |
| P172 | EU | MEX | M | *CYBB* | ex. 7 | c.742dupA | p.Ile248Asnfs*36 | *Insertion* | Hemi | X91^0^ | Alive | [13] |
| P173 | EV | MEX | M | *CYBB* | ex. 2 | c.141+1G>T | del. ex. 2? (p.Leu16_Gly47del) | *Splicing* | Hemi | X91^−^ | Alive | [13, 3, 50] |
| P174 | EW | MEX | M | *CYBB* | ex. 7 | c.752G>A | p.Trp251* | *Nonsense* | Hemi | X91^0^ | Alive | [3, 50] |
| P175 | EX | MEX | F | *NCF2* | ex. 2 / ex. 2 | c.55_63del / c.74C>A | p.Lys19_Asp21del / p.Ala25Asp | *Deletion* / *Missense* | Comp. | A67^0^ / A67^?^ | Alive | [13, 4] |
| P176 | EY | MEX | M | *CYBB* | del. ex. 1_13 | del. ex. 1_13 | − | *Large del. (≥1 ex.)* | Hemi | X91^0^ | Alive | [13] |
| P177 | EZ | MEX | M | *CYBB* | ex. 10 | c.1275C>A | p.Tyr425* | *Nonsense* | Hemi | X91^?^ | Alive | [3, 50] |
| P178 | FA | MEX | M | *CYBA* | ex. 5 | c.354C>A | p.Ser118Arg | *Missense* | Homo | A22^0^ | Alive | [4] |
| P179 | FBsi | MEX | M | *CYBB* | ex. 6 | c.580del | p.Thr194Profs*20 | *Deletion* | Hemi | X91^0^ | Alive | [13, 33] |
| P180 | FC | MEX | M | *NCF1* | ex. 2 | c.75_76delGT | p.Tyr26Hisfs*26 | *Deletion* | Homo | A47^0^ | Alive | [4] |
| P181 | FD | MEX | M | *CYBB* | ex. 10 | − | p.GIy412Val | *Missense* | Hemi | X91^−^ | Dead | [13] |
| P182 | FE | MEX | M | UnK. | − | − | − | − | − | − | Alive | − |
| P183 | FF | MEX | M | UnK. | − | − | − | − | − | − | Alive | − |
| P184 | FG | MEX | M | *CYBB* | ex. 7 | c.676C>T | p.Arg226* | *Nonsense* | Hemi | X91^0^ | Dead | [13, 3, 50] |
| P185 | CPc | MEX | M | *CYBA* | ex. 1 | c.4_24del21 | p.Gly2_Met8del | *Deletion* | Homo | A22^0^ | Alive | [13] |
| P186 | FH | MEX | F | *NCF2* | ex. 2 | c.229C>T | p.Arg77* | *Nonsense* | Homo | A67^0^ | Alive | [4] |
| P187 | FIc | MEX | M | *CYBB* | ex. 1 | c.13delG | p.Ala5Leufs*2 | *Deletion* | Hemi | X91^−^ | Alive | [13] |
| P188 | FIc | MEX | M | *CYBB* | ex. 1 | c.13delG | p.Ala5Leufs*2 | *Deletion* | Hemi | X91^−^ | Dead | [13] |
| P189 | FJ | MEX | M | *CYBB* | del. ex. 1_3 | del. ex. 1_3 | − | *Large del. (≥1 ex.)* | Hemi | X91^0^ | Alive | [13, 3] |
| P190 | FK | MEX | M | *CYBB* | ex. 2 | c.80_83delTCTG | p.Val27Glyfs*33 | *Deletion* | Hemi | X91^0^ | Alive | [13, 33, 3] |
| P191 | FL | MEX | M | UnK. | − | − | − | − | − | − | Alive | − |
| P192 | FM | MEX | M | *NCF2* | in. 3 / ex. 1 | c.366+1G>A / c.124A>C | del. ex. 3 e 4 / p.Asn42His | *Splicing / Missense* | Comp. | A67^0^ / A67^?^ | Alive | [4 / ND] |
| P193 | DGn | MEX | M | *CYBB* | ex. 9 | c.978delT | p.Phe326Leufs*17 | *Deletion* | Hemi | X91^?^ | Dead | ND |
| P194 | FN | MEX | F | *NCF2* | ex. 1 | c.137T>G | p.Met46Arg | *Missense* | Homo | A67^0^ | Alive | [13, 49] |
| P195 | FO | MEX | M | UnK. | − | − | − | − | − | − | Alive | − |
| P196 | FP | MEX | M | *CYBB* | ex. 3 | c.207delinsTT | − | *Indel* | Hemi | X91^0^ | Alive | [13, 49] |
| P197 | FQ | MEX | M | *CYBB* | del. ex. 1_3 | del. ex. 1_3 | − | *Large del. (≥1 ex.)* | Hemi | X91^0^ | Alive | [13, 3] |
| P198 | EJ | MEX | M | *CYBB* | ex. 9 | c.1085C>G | p.Thr362Arg | *Missense* | Hemi | X91^?^ | Alive | [3] |
| P199 | FR | MEX | M | *CYBB* | in. 8 | c.898-1G>A | del. ex. 9? (p.Val300_Pro383del) | *Splicing* | Hemi | X91^0^ | Dead | [13, 49] |
| P200 | FS | MEX | M | *CYBB* | del. ex. 3 | c.252G>A | del. ex. 3 (p.Ser48_Ala84del) | *Splicing* | Hemi | X91^0^ | Alive | [3, 50] |
| P201 | CSu | MEX | M | *CYBB* | ex. 9 | c.1016C>A | p.Pro339His | *Missense* | Hemi | X91^0^ | Alive | [13, 3, 50] |
| P202 | FT | MEX | M | UnK. | − | − | − | − | − | − | Alive | − |
| P203 | FU | MEX | M | *CYBB* | ex. 12 | c.1521_1523del | p.Lys508del | *Missense* | Hemi | X91^?^ | Alive | [13, 3] |
| P204 | FV | MEX | M | *CYBB* | ex. 12 | c.1508C>A | p.Thr503Lys | *Missense* | Hemi | X91^+^ | Alive | [13] |
| P205 | FW | MEX | F | *CYBB* | ex. 7 | c.676C>T | p.Arg226* | *Nonsense* | Hetero | X91^0^ # | Dead | [67, 50] |
| P206 | FX | MEX | M | *CYBB* | Prom. | c.-65C>T | − | *Deletion* | Hemi | X91^0^ | Alive | [13, 3, 50] |
| P207 | FY | MEX | M | *CYBB* | ex. 9 | c.1038delT | p.Glu347Argfs*39 | *Deletion* | Hemi | X91^0^ | Dead | [13, 3, 50] |
| P208 | FZ | MEX | M | *CYBB* | ex. 10 | c.1234G>A | p.Gly412Arg | *Missense* | Hemi | X91^−^ | Dead | [13, 3] |
| P209 | GA | MEX | M | *CYBA* | ex. 4 | c.287T>C | p.Leu96Pro | *Missense* | Homo | A22^0^ | Alive | [13] |
| P210 | FBsi | MEX | M | *CYBB* | ex. 6 | c.580del | p.Thr194Profs*20 | *Deletion* | Hemi | X91^0^ | Dead | [13, 33] |
| P211 | FBsi | MEX | M | UnK. | − | − | − | − | − | − | Alive | − |
| P212 | GB | MEX | F | UnK. | − | − | − | − | − | − | Dead | − |
| P213 | GC | MEX | M | UnK. | − | − | − | − | − | − | Alive | − |
| P214 | GD | MEX | M | *CYBB* | ex. 7 | c.676C>T | p.Arg226* | *Nonsense* | Hemi | X91^0^ | Alive | [13, 3, 50] |
| P215 | GE | CHI | F | *NCF2* | ex. 8-9 | Duplication | − | Duplication | Homo | A67^?^ | Dead | ND |
| P216 | GF | CHI | M | *CYBB* | ex. 7 | c.676C>T | p.Arg226* | *Nonsense* | Hemi | X91^0^ | Alive | [13, 3, 50] |
| P217 | GG | CHI | M | *CYBB* | ex. 7 | c.781C>T | p.Gln261* | *Nonsense* | Hemi | X91^0^ | Alive | [3, 50] |
| P218 | GH | CHI | M | UnK. | − | − | − | − | − | − | Alive | − |
| P219 | GI | CHI | M | *CYBB* | in. 6 | c.675-2A>G | Splicing site | *Splicing* | Hemi | X91^?^ | Alive | ND |
| P220 | GJ | CHI | F | UnK. | − | − | − | − | − | − | Dead | − |
| P221 | GK | CRC | M | UnK. | − | − | − | − | − | − | Alive | − |
| P222 | GL | CRC | M | UnK. | − | − | − | − | − | − | Alive | − |
| P223 | GM | CRC | F | *NCF1* | ex. 2 | c.75_76delGT | p.Tyr26Hisfs*26 | *Deletion* | Homo | A47^0^ | Alive | [4] |
| P224 | GN | CRC | M | *CYBB* | ex. 7 | c.725_726delCA | p.Thr242Serfs*2 | *Deletion* | Hemi | X91^?^ | Alive | [49] |
| P225 | GO | CRC | M | *CYBB* | ex. 1 | c.34delA | p.Ile12Phefs*10 | *Deletion* | Hemi | X91^?^ | Alive | ND |
| P226 | GP | ARG | M | *CYBB* | ex. 9 | c.1096T>G | p.Trp361Gly | *Missense* | Hemi | X91^?^ | Alive | ND |
| P227 | GQc | ARG | M | *CYBB* | ex. 13 | c.1598_1600delGAG | p.Gly533del | *Deletion* | Hemi | X91^−^ | Alive | [3] |
| P228 | GQc | ARG | M | *CYBB* | ex. 13 | c.1598_1600delGAG | p.Gly533del | *Deletion* | Hemi | X91^−^ | Dead | [3] |
| P229 | GR | ARG | M | *CYBB* | ex. 2 | Del. ex. 2 | del. ex. 2 (p.Leu16_Gly47del) | *Splicing* | Hemi | X91^0^ | Alive | [3] |
| P230 | GS | PAR | M | UnK. | − | − | − | − | − | − | Alive | − |
| P231 | GT | PAR | M | UnK. | − | − | − | − | − | − | Dead | − |
| P232 | GU | PAR | M | UnK. | − | − | − | − | − | − | Alive | − |
| P233 | GV | PAR | M | UnK. | − | − | − | − | − | − | Alive | − |
| P234 | GW | PER | M | *CYBB* | ex. 9 | c.1081T>C | p.Trp361Arg | *Missense* | Hemi | X91^0^ | Alive | [3] |
| P235 | GX | PER | M | *CYBB* | ex. 5 | c.388C>T | p.Arg130* | *Nonsense* | Hemi | X91^0^ | Alive | [3, 50] |
| P236 | GY | PER | M | *CYBB* | in. 9 | c.1152-1G>A | Splicing site | *Splicing* | Hemi | X91^0^ | Dead | [3] |
| P237 | GZ | URU | M | *CYBB* | in. 3 | c.253-8A>G | Splicing site | *Splicing* | Hemi | X91^0^ | Alive | [3, 50] |
| P238 | H | URU | M | UnK. | − | − | − | − | − | − | Alive | − |

*Fam.*, Family; *Count.*, Country; *Mut.*, Mutation; *Zygo.*, Zygosity; *Hemi.*, hemizygosity; *Homo.*, homozygosity; *Comp.*, compound heterozygosity; *ex.*, exon; *in.*, intron; *Express.*, Expression; *Del.*, Deletion; *Prom.*, promoter; *UnK*., Unknown; *ND*, Not described. The lowercase letter in the family coding represents the degree of kindred: *c*, cousin; *si*, siblings; *u*, uncle; *n*, niece/nephew; *so*, son; *m*, mother; MEX, Mexico; BRA, Brazil; CHI, Chile; CRC, Costa Rica; ARG, Argentina; PAR, Paraguay; PER, Peru; URU, Uruguay. In the “Type” column, the letter X corresponds to X-linked inheritance and the letter A corresponds to autosomal recessive forms, the number corresponds to the affected protein. Superscript information corresponds to the final effect on the expression or function of the protein: (-) lower expression protein/function; (+) normal protein expression, but with impaired function; (zero “0”) absence of expression and/or function; (?) lack of information regarding the expression and/or function of the affected protein. #, patients with possible distorted X chromosome inactivation (Skewed X-inactivation). McLeod phenotype with a large deletion on the X chromosome (X-linked) that involve some genes such as *CYBB* and *XK* genes (encodes Kx antigen). Nomenclature of mutations according to the recommendations of the American College of Medical Genetics and Genomics (ACMG) and the Human Genome Variation Society (HGVS) [35]
